# Dense 4D nanoscale reconstruction of living brain tissue

**DOI:** 10.1038/s41592-023-01936-6

**Published:** 2023-07-10

**Authors:** Philipp Velicky, Eder Miguel, Julia M. Michalska, Julia Lyudchik, Donglai Wei, Zudi Lin, Jake F. Watson, Jakob Troidl, Johanna Beyer, Yoav Ben-Simon, Christoph Sommer, Wiebke Jahr, Alban Cenameri, Johannes Broichhagen, Seth G. N. Grant, Peter Jonas, Gaia Novarino, Hanspeter Pfister, Bernd Bickel, Johann G. Danzl

**Affiliations:** 1 Institute of Science and Technology Austria (ISTA), Klosterneuburg, Austria; 2 School of Engineering and Applied Sciences, Harvard University, Cambridge, MA USA; 3 Leibniz-Forschungsinstitut für Molekulare Pharmakologie, Berlin, Germany; 4 Genes to Cognition Program, Centre for Clinical Brain Sciences, University of Edinburgh, Edinburgh, UK; 5 Simons Initiative for the Developing Brain (SIDB), Centre for Discovery Brain Sciences, University of Edinburgh, Edinburgh, UK; 6 Present Address: Core Facility Imaging, Medical University of Vienna, Vienna, Austria; 7 Present Address: Department of Computer Science, Boston College, Boston, MA USA; 8 Present Address: Allen Institute for Brain Science, Seattle, WA USA; 9 Present Address: In-Vision Technologies, Guntramsdorf, Austria

**Keywords:** Super-resolution microscopy, Cellular neuroscience

## Abstract

Three-dimensional (3D) reconstruction of living brain tissue down to an individual synapse level would create opportunities for decoding the dynamics and structure–function relationships of the brain’s complex and dense information processing network; however, this has been hindered by insufficient 3D resolution, inadequate signal-to-noise ratio and prohibitive light burden in optical imaging, whereas electron microscopy is inherently static. Here we solved these challenges by developing an integrated optical/machine-learning technology, LIONESS (live information-optimized nanoscopy enabling saturated segmentation). This leverages optical modifications to stimulated emission depletion microscopy in comprehensively, extracellularly labeled tissue and previous information on sample structure via machine learning to simultaneously achieve isotropic super-resolution, high signal-to-noise ratio and compatibility with living tissue. This allows dense deep-learning-based instance segmentation and 3D reconstruction at a synapse level, incorporating molecular, activity and morphodynamic information. LIONESS opens up avenues for studying the dynamic functional (nano-)architecture of living brain tissue.

## Abstract (web summary)

A combination of gentle stimulated emission depletion microscopy imaging and deep-learning-based improvements in signal-to-noise ratio enables high-resolution reconstruction of neuronal architecture in living tissue.

## Main

Brain computation and information storage are intimately linked to the structure of a synaptic network of ~86 billion neurons^1^ in humans. To address how this crowded and complex tissue’s architecture, connectivity and functional activity evolve over time, one would ideally employ a technology that enables imaging and in silico reconstructing living brain tissue.

Electron microscopy (EM) reconstruction offers the most detailed insights into brain architecture by tracing all neuronal structures and determining connectivity with single-synapse accuracy, thus unraveling ‘connectomes’^2–10^; however, this is limited to static representations, whereas specific molecular labeling requires correlative approaches^11^. A light-microscopy-based technology for tissue reconstruction would enable observation of structural dynamics in living systems. The intricacy of brain tissue demands a 3D super-resolution approach^12–15^, as reconstruction is limited by the lowest-resolution direction (typically along the optical axis; *z* direction). Conventional (diffraction-limited) microscopy is unsuitable, with its best-case lateral resolution of ~half the wavelength of employed light and axial resolution as poor as ~1,000 nm for tissue-compatible objective lenses and far-red excitation.

Here we introduce dense reconstruction of living brain tissue at a single-synapse level. Rather than aiming at connectomic circuit investigation, our technology unlocks morphodynamics in nanoscale-resolved 3D reconstruction, while simultaneously accessing molecular and functional information. We developed an integrated optical/machine-learning technology breaking the intertwined limitations for 3D-resolving power, signal-to-noise ratio (SNR), speed and light burden in live super-resolution imaging. We based our technology on stimulated emission depletion^12,16^ (STED) microscopy. Here, a light pattern turns off fluorophores except those near its intensity minimum and positions are queried sequentially. Unlike visualization of protein distributions or sparse cells^17^, dense tissue reconstruction requires unbiased delineation of all cells. We therefore built on super-resolution shadow imaging^18^, where extracellularly applied fluorophores^19^ reveal cellular structures and arrangements^20–22^ and photobleached fluorophores are replenished by diffusion. Despite these advantages, synapse-level reconstruction of living brain tissue has been elusive. The square-root dependence of resolution on applied STED power^23^ and 3D-sampling steps of a few tens of nanometers impose a heavy cost of light burden to increase 3D resolution^24^. Together with optical imperfections causing progressive signal loss at higher resolution, these factors limit 3D resolution and SNR^25^.

**Fig. 1:**
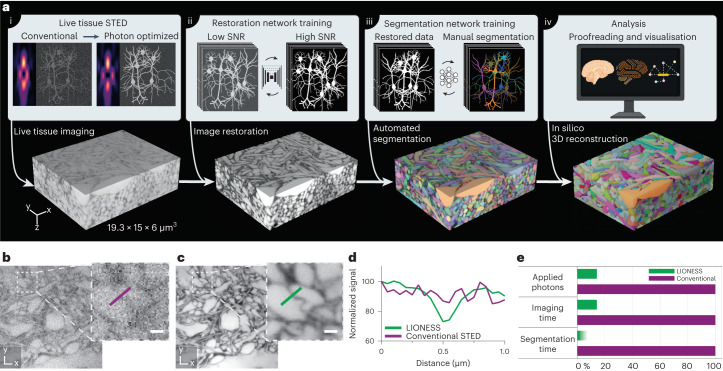
LIONESS enables dense reconstruction of living brain tissue. **a**, LIONESS technology exemplified in living human cerebral organoid. Optical improvements, deep-learning training and analysis (top) flow into individual processing steps (bottom). (i) Near-infrared STED with light patterns for improved effective point-spread-function in tissue. (ii) Deep neural network training on paired low-exposure, low-SNR and high-exposure, high-SNR 3D super-resolved volumes recorded in extracellularly labeled tissue. (iii) Deep 3D-segmentation network training with manually annotated data. (iv) Postprocessing. **b**, Conventional STED imaging in CA1 neuropil of extracellularly labeled organotypic hippocampal slice culture with phase modulation patterns for lateral (*xy*) plus axial (*z*)-resolution increase. **c**, Same region imaged in LIONESS mode with tissue-adapted STED patterns (4π-helical plus π-top-hat phase modulation), modified detector setup and deep-learning-based image restoration. STED power and dwell time were identical in **b** and **c**. The images are representative of *n* = 3 technical replicates from two samples. Scale bars, 500 nm. **d**, Line profiles across a putative synaptic cleft as indicated in **b** and **c**. **e**, Schematic comparison of LIONESS imaging with conventional STED imaging (based on the parameters used in restoration network training) in terms of light exposure and imaging time, as well as the reduction in segmentation time by automated over manual segmentation. The shading indicates that reduction in segmentation time by deep learning depends on sample complexity. LIONESS lookup tables are linear and inverted throughout, ranging from black (maximum photon counts extracellularly) to white.

We therefore modified STED for improved SNR and isotropically super-resolved tissue imaging, coupled with a two-stage deep-learning strategy. Stage one leveraged information on sample structure from numerous separate, previous measurements to reduce light burden and imaging time without sacrificing resolution, and hence enabled live-tissue-compatible volumetric super-resolution imaging. Stage two was adapted from EM connectomics to translate our volumetric live-imaging data into nanoscale-resolved instance segmentations. We termed this technology LIONESS (live information-optimized nanoscopy enabling saturated segmentation) (Fig. 1a)^26^. LIONESS unites live imaging with unbiased nanoscale reconstruction, extending tissue analysis with information on morphological dynamics, molecular identities and neuronal activity.

## Results

### Isotropic high-SNR STED in tissue

**Extended Data Fig. 1:**
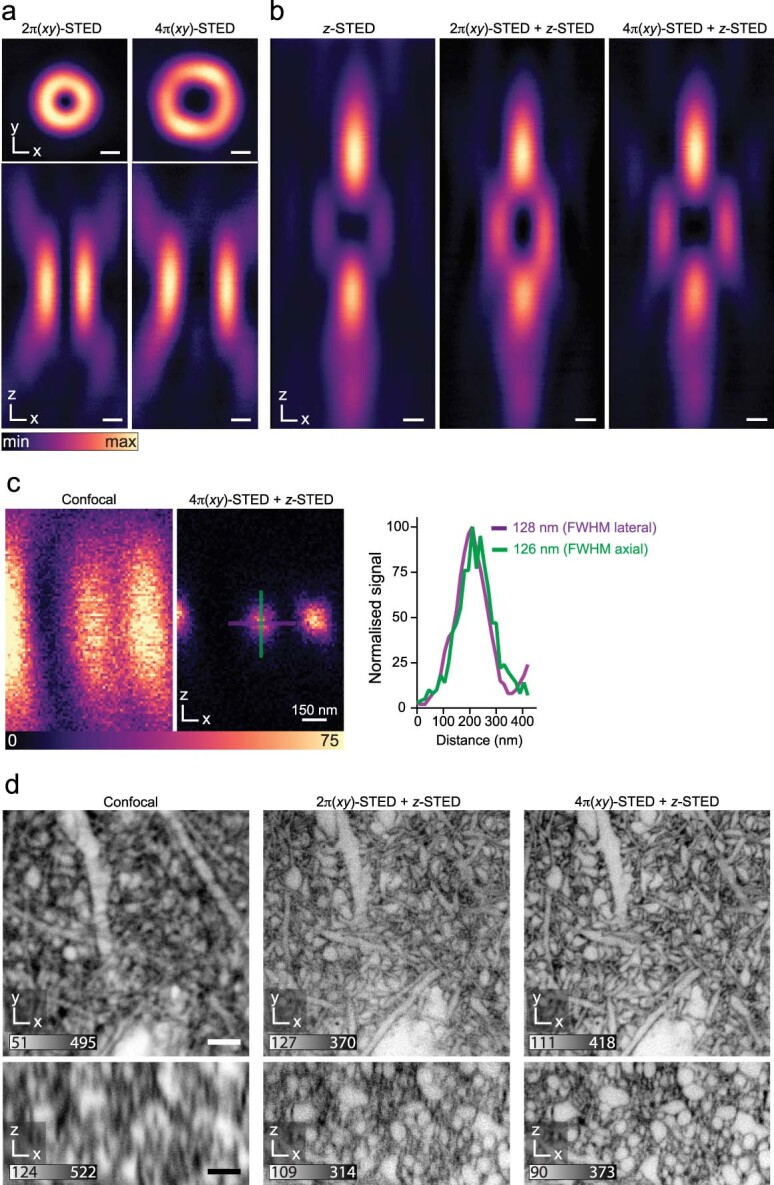
Tissue-optimized STED. **a, b**, STED light intensity distributions in the focal region, measured via backscattering from a 150 nm diameter gold nanosphere. **a**, Lateral (*top*) and axial (*bottom*) sections for 2π- and 4π-helical phase modulation. Scale bars: 250 nm. **b**, Axial sections of the π-top-hat phase modulated *z*-STED pattern (*left*), an incoherent superposition of 2π(*xy*)- and *z*-STED patterns (*middle*) and of 4π(*xy*)- and *z*-STED patterns (*right*). Power distribution between the *z*- and *xy*-STED patterns in the superpositions was 80% vs. 20%. Scale bars: 250 nm. **c**, Axial scan of 40 nm diameter Crimson fluorescent beads (Abberior) in confocal mode (left image) and with STED employing combined 4π(*xy*)- and *z*-STED patterns (right image) with the same power distribution as before. Scale bar: 250 nm. Profiles (2 pixel width) along the lines in lateral and axial directions as indicated in the image. Images are representative of n = 2 experiments. **d**, Extracellularly labeled neuropil in organotypic hippocampal slices. Orthogonal planes in *xy*- and *xz*-direction for diffraction-limited confocal (*left*), classical 2π-helical and π-top-hat phase modulation (*middle*), and combination of 4π-helical plus π-top-hat modulation for isotropic resolution with improved quenching of excitation outside the central STED intensity minimum (*right*). The 4π-helical pattern also facilitated robust in-tissue co-alignment of intensity minima. The images are representative of n = 3 technical replicates in two samples. Scale bars: 2 µm. Raw data with linear, inverted color scale. Numbers in greyscale bars refer to raw photon counts.

**Extended Data Fig. 2:**
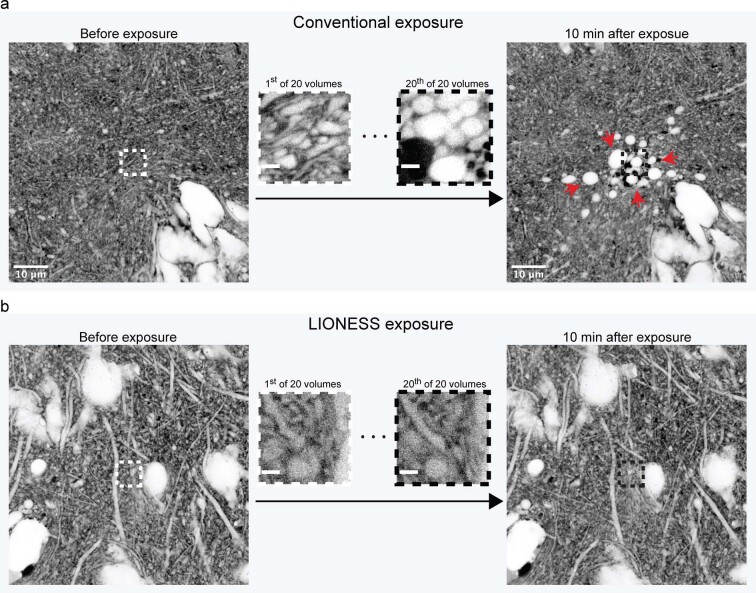
Live-tissue compatibility. **a**, Confocal overview images in an organotypic hippocampal slice culture before (*left*) and 10 minutes after (*right*) scanning a volume in the center (5 × 5 × 2.5 µm^3^) 20 times in high-photon load STED mode (70 µs voxel integration time). Central images are single planes of the first and last STED volume acquired. Red arrows indicate blebbing and disintegrating cells. **b**, Confocal overview images of a different region before (*left*) and 10 minutes after (*right*) scanning a volume in the center (5 × 5 × 2.5 µm^3^) 20 times using LIONESS parameters (10 µs voxel integration time). Central images are single planes of the first and last LIONESS volume acquired. The images are representative of n = 4 experiments recorded across two biological samples. Scale bars: confocal: 10 µm, STED: 1 µm.

We chose near-infrared STED (775 nm) for the highest STED performance and reduced tissue absorption and scattering over visible light^24,27^. We screened for cell-impermeant fluorophores to label the extracellular space (ECS) selectively and identified suitable hydrophilic, anionic high-performance STED labels (Supplementary Fig. 1). Aiming for isotropic STED resolution, we overlapped classical 2π-helical and π-top-hat phase modulation patterns for lateral (*xy*) and predominantly axial (*z*) STED resolution increase^25^, respectively, mitigating (spherical) aberrations on the sensitive *z*-STED pattern using a silicone immersion objective with correction collar and partially pre-compensating with a spatial light modulator (SLM); however, as expected, the combined intensity minimum was highly susceptible to aberrations and imperfect spatial overlap in tissue. We therefore replaced the 2π(*xy*)-pattern with a helicity-2 mode (4π-helical phase modulation)^28^. The shallower rise and broader distribution of the 4π(*xy*)-STED pattern facilitated in-tissue alignment and quenching of ‘sidelobe’ fluorescence insufficiently silenced by the *z*-STED pattern (Extended Data Fig. 1a–c). This combination yielded isotropic resolution and crucially, improved spatial definition of on/off contrast with substantially enhanced distinction of cellular structures (Extended Data Fig. 1d). We achieved a further slight SNR improvement with higher excitation using two parallel single photon detectors (Supplementary Fig. 2). Mitigating spherical aberrations as before resulted in high imaging performance in 8–10-µm thick regions up to ~50 µm depth, limited by distortion and scattering of the STED beam (Supplementary Fig. 3). Similarly, performance was best in the central ~25 µm of the objective field of view. To delineate narrow spaces between cells with extracellular label in 3D and detect fluorescence modulation produced by thin cellular processes with sufficient SNR for segmentation, we integrated photons for 70 µs per 50 × 50 × 50 nm^3^ voxel and dialed in isotropic resolution of ≲130 nm (Extended Data Fig. 1c); however, this was too harsh and too slow for volumetric imaging of living tissue, causing substantial photodamage (Extended Data Fig. 2a).

### Low-exposure, high-speed STED

**Extended Data Fig. 3:**
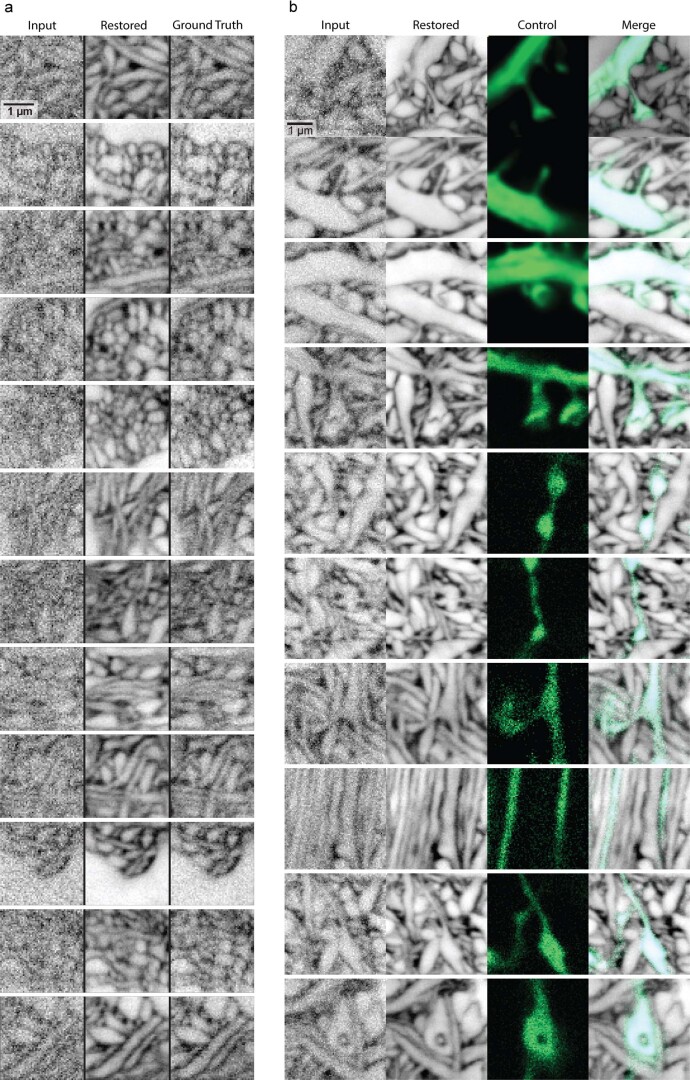
Validation of image restoration. **a**, Twelve example areas of raw low-exposure input, image-restored artificial neural network prediction, and high-SNR ‘ground truth’ from one imaging volume not included in the network training data. Neuropil in extracellularly labeled organotypic hippocampal slice culture, imaged with the same STED parameters as used in the restoration network training (including π-top-hat plus 4π helical phase modulation STED patterns at 80/20 power split) for both the low-exposure measurement (10 µs voxel dwell time) and the high-exposure ‘ground truth’ measurement (70 µs voxel dwell time). **b**, Ten example areas of raw low-exposure input, image-restored artificial neural network prediction, positively labeled control (*Thy1-eGFP*, confocal), and overlay of image-restored network output and control in extracellularly labeled organotypic hippocampal slice culture. Images are from n = 5 datasets recorded across 4 biological samples. Scale bars: 1 µm. Images except for positively labeled control displayed with inverted lookup table. Maximum intensity projections spanning 150 nm. Data used in validation were not part of restoration network training.

We sought strategies to reduce light burden and imaging time while augmenting SNR. We thus recorded low-exposure STED data at high speed and deployed deep-learning image restoration, retrieving previous information on sample structure. We trained a convolutional neural network^29^ (Supplementary Fig. 4a) on paired low- and high-SNR imaging volumes from extracellularly labeled mouse organotypic hippocampal slice cultures and the alveus region of acutely prepared mouse hippocampus. These were sampled at high SNR with 70 µs voxel dwell time, from which we set aside photon counts of the first 10 µs as low-SNR training input data, ensuring voxel-exact correspondence of represented sample structures. We then applied the trained model to unseen data to predict high-SNR images from low-exposure input data. To evaluate whether the prediction represented biological structures faithfully in the context of cellular segmentation, per-voxel probabilistic uncertainty measures and ensemble disagreement between independently trained networks^29^ were of limited utility (Supplementary Fig. 4b). Therefore, we compared predictions with paired high-SNR measurements, using data not included in the training (Extended Data Fig. 3a) and with a sparse positive cellular label (Extended Data Fig. 3b). This indicated that inaccuracies at the voxel level did not negatively impact definition of cellular structures.

Repeated volumetric imaging in this low-exposure mode left cells intact, whereas they disintegrated when aiming at similar resolution and SNR with conventional high-photon load STED (Extended Data Fig. 2). Our scheme reduced photon load by 86%. Contrasting with current techniques^25^ for reducing STED exposure^30,31^ and photobleaching^32,33^, it also accelerated acquisition sevenfold. Integrating labeling, optimizations for in-tissue isotropically resolving super-resolution imaging, low-exposure data collection and computational image restoration resulted in a LIONESS imaging regime with substantial quality gain over conventional STED imaging for given live-tissue-compatible STED light exposure (Fig. 1b–e). Together, this yielded volumetric light-microscopy data of living nervous tissue suitable for segmentation.

### In silico reconstruction

Manual annotation in a small LIONESS volume showed that dense live-tissue reconstruction was in principle feasible; however, segmenting a ~400-µm^3^ volume of living hippocampal tissue took a trained segmenter ~450 h (Supplementary Fig. 5). We therefore implemented a second deep neural network, adapting algorithms and software from EM connectomics^34,35^, for automated segmentation (Supplementary Software). We initially trained the network on a subvolume of the manually annotated data (~285 µm^3^, using the other part for validation) and applied it with watershed postprocessing to larger volumes harboring additional structural diversity (CA1 and dentate gyrus (DG) neuropil in hippocampal slice cultures and alveus in acutely prepared hippocampi). We proofread the output (inspected automatically generated segments in relation to the LIONESS imaging data) and manually corrected segments according to human judgment. Feeding this back extended the training volume to ~800 µm^3^, yielding a segmentation model with enhanced prediction quality.

We chose living human cerebral organoids^36^, a powerful model for brain development and disease mechanisms, as first specimen for automated reconstruction. LIONESS enabled comprehensive reconstruction (Fig. 1a) and, in such samples with moderately complex structure, required minor proofreading intervention. LIONESS revealed contextual information missing with sparse labeling, including how an axonal growth cone interacted with neighboring structures (Supplementary Fig. 6). The gain in throughput from automated over manual segmentation was substantial, with data acquisition (140 s), image restoration (10 s) and automated segmentation (~40 min) taking <45 min excluding data inspection and proofreading (Fig. 1e). Manual segmentation would require an estimated ~860 person-hours for this dataset (1,737 µm^3^). Extracting the space not occupied by cellular segments allowed reconstructing the ECS (225 µm^3^ or 13% in this volume; Supplementary Fig. 7).

**Extended Data Fig. 4:**
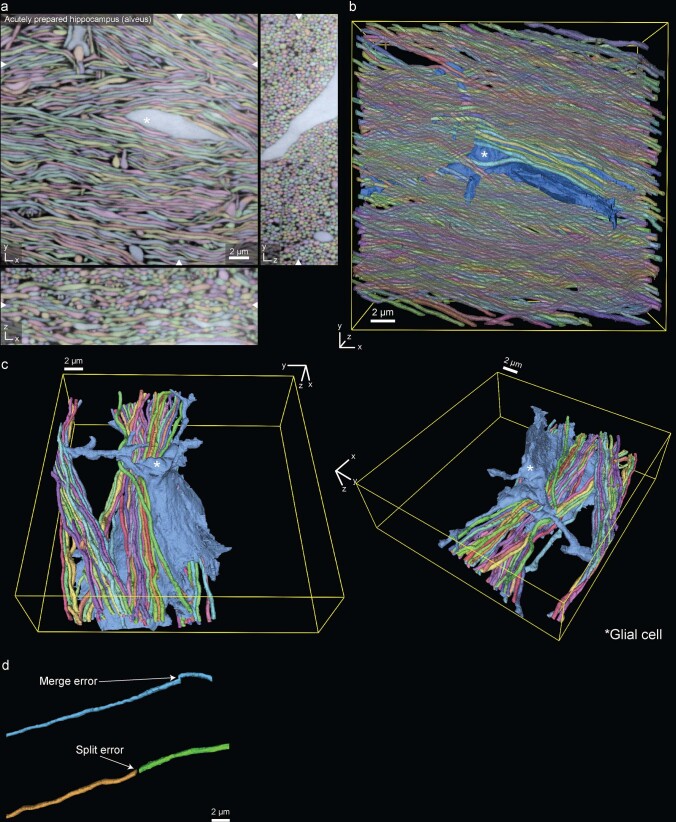
Reconstruction in living hippocampal alveus. **a**, Three orthogonal planes from a fully segmented LIONESS volume in the alveus region of an acutely prepared mouse hippocampus. The white asterisk indicates a glial cell stretching through dense axons. White arrowheads at image edges indicate the position of orthogonal *xy*-, *yz*- or *xz*-views. Scale bar: 2 µm. **b**, 3D-rendering of a subset of structures from the same dataset as shown in panel a. **c**, 3D reconstruction of the glial cell marked in panel a and selected axons, viewed from two different angles. **d**, Examples of error types after automated segmentation.

Next, we chose the alveus of intact, acutely dissected mouse hippocampi, a region densely packed with thin neurites for LIONESS reconstruction highlighting the thin, individually resolved axons running in various orientations and interacting with glial cells (Extended Data Fig. 4a–c and Supplementary Videos 1 and 2). Such structurally comparatively homogeneous regions also required little intervention during proofreading. Approximately 45 corrections per mm axon length were necessary, with false splits being the dominant error type (Extended Data Fig. 4d). This showed that comprehensive structural segmentation of living nervous tissue is feasible. Furthermore, deep-learning segmentation models were applicable across tissue preparations.

### Validation of segmentation

To test the potential and limitations for analysis of complex specimens, we collected imaging volumes from highly interwoven neuropil in organotypic hippocampal slices. We first focused on assigning dendritic spines to dendrites, as the fine connecting necks are among the thinnest of neuronal structures.

**Fig. 2:**
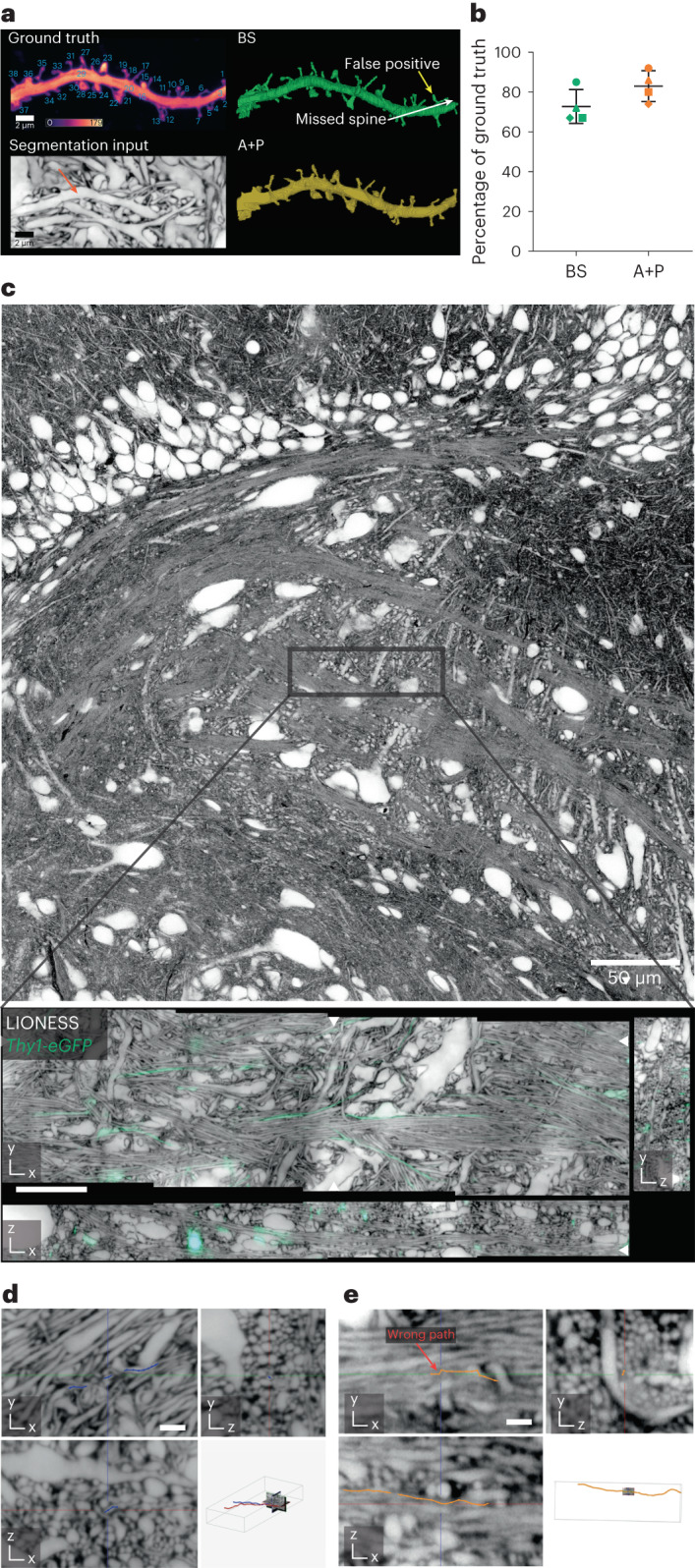
Validation of segmentation. **a,** Maximum intensity projection of a dendrite in organotypic hippocampal slice culture labeled by cytosolic eGFP expression (*Thy1-eGFP* mouse line, confocal) (top left). The positive label served as ground truth for spine detection, with individual spines numbered. Calibration bar represents raw photon counts. Plane from isotropically super-resolved, volumetric LIONESS acquisition used as source data for segmentation with arrow indicating the same dendrite in the extracellularly labeled tissue (maximum intensity projection spanning 150 nm) (bottom left). Fully manual spine detection from LIONESS imaging data by a segmenter blinded to the eGFP channel (BS, blinded segmenter) (top right). Examples of missed and false-positive spines are indicated by white and yellow arrows, respectively. Spine detection in 3D reconstruction after automated segmentation and proofreading of LIONESS imaging data by the experimenter who recorded the data (automated plus proofreading, A + P) (bottom right). As this person was not blinded to the eGFP channel, this indicates which spines can be retrieved from LIONESS but does not serve as an independent control. **b**, Percentage of correctly assigned spines from the automated plus proofread (A + P, orange) and manual (BS, green) segmentations relative to the total number of spines in the positively labeled ground truth with mean and s.d. for *n* = 4 different dendrite stretches originating from three different organotypic hippocampal slices (images for remaining datasets are shown in Supplementary Fig. 8). Correctly identified spines were counted as +1, false positives as −1. In two cases out of 129 the manual tracer identified the spine but omitted the spine head. These were counted as +0.5. **c,** Confocal overview in organotypic hippocampal slice with a band of DG cells in the top part and the start of CA3c in the center (top). Orthogonal planes from LIONESS volume at the position indicated in the top panel, including sparse eGFP expressing axons (*Thy1-eGFP*, confocal) used as ground truth for tracing (bottom). White arrowheads at image edges indicate position of corresponding orthogonal planes. Scale bar, 10 µm. LIONESS images are maximum intensity projections spanning 150 nm. **d**, Magnified view of three orthogonal planes from a LIONESS imaging volume in an organotypic hippocampal brain slice with example of a correctly traced axon (blue line with dots, manually generated in WebKnossos by a tracer blinded to the eGFP channel). Scale bar, 1 µm. Position of subvolume relative to full imaging volume with two traced axons in red and blue (bottom right). **e**, Example of an error in tracing (orange line with dots), marked with red arrow. The images in **c**–**e** are representative for tracing a total of nine axons in *n* = 3 different cultured brain slices from *Thy1-eGFP* mice. Scale bar, 1 µm.

Light microscopy allows using an additional color channel to obtain ‘ground truth’ on the structure of sparsely highlighted neurons, providing ‘end-to-end’ validation independent of the evaluated LIONESS data. Focusing on regions where labeled dendrites were sufficiently spaced to avoid signal overlap from neighboring structures^37^, cytosolic enhanced green fluorescent protein (eGFP) expression revealed all dendritic spines on a dendrite (Fig. 2a,b and Supplementary Fig. 8). We read out intracellular eGFP with confocal microscopy, which was adequate for detecting spines, as required here, but would be unsuitable for characterizing their 3D shape. From LIONESS data alone, without automated segmentation, a neuroscientist blinded to eGFP correctly assigned 73% (±8.3%, mean ± s.d.) of spines in four dendrite stretches (from three biological replicates; 129 spines total, 34 missed and 2 false positive). Applied to the same datasets, the artificial network often segmented and correctly connected spines to the respective dendrite or classified spines as separate (orphan) segments that could be unambiguously assigned to a dendrite. The experimenter who collected the data performed proofreading of automated segmentation output and correctly attached 83% (±8.0%, mean ± s.d.; 129 spines total, 20 missed and 0 false positive) of spines. There was no obvious correspondence between locations of wrongly assigned spines and local image restoration uncertainty (Supplementary Fig. 9).

Similarly, we tested to what extent particularly challenging structures (thin axons) could be resolved and traced, demarcating limitations in traceability. As expected from the alveus data above (Extended Data Fig. 4), prominent mossy fiber axons in the CA3 region were conspicuous (Fig. 2c); however, resolution and SNR were insufficient for tracing thin, tortuous axons in complex neuropil and we found regions where such structures coalesced (Fig. 2d,e). Specifically, we evaluated nine axons (total length 565 µm) in three datasets from hippocampal slice cultures with sparse eGFP ground truth. A blinded segmenter correctly traced 302 µm, with 33.6 µm (±18.2 µm s.d.) mean error-free segment length. The longest correctly traced segment spanned 74.6 µm, traversing the dataset fully. Overall, this demonstrated applicability of LIONESS for dense nanoscale analysis of neuropil architecture, whereas connectomic reconstruction (comprehensive tracing and assignment of all synaptic connections) would require improving the accuracy further.

### Connectivity reconstruction

**Fig. 3:**
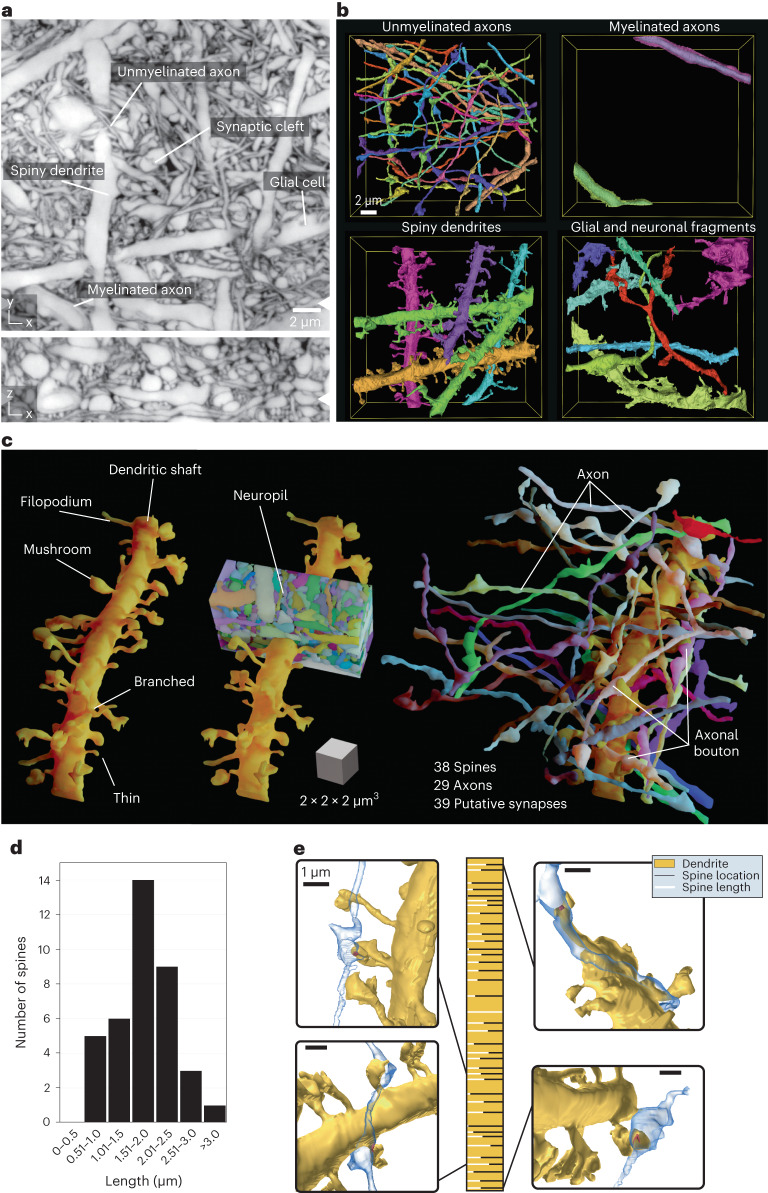
Connectivity reconstruction in live hippocampus. **a**, Orthogonal planes from an isotropically super-resolved LIONESS volume in *xy* and *xz* directions in neuropil of the DG in organotypic hippocampal slice culture from a *Prox1-cre::Ai95* mouse. White arrowheads at image edges indicate position of corresponding orthogonal planes. The image is representative of *n* > 20 repetitions. Scale bar, 2 µm. LIONESS images are maximum intensity projections spanning 150 nm. **b**, 3D reconstructions of example cellular structures extracted from a. **c**, 3D reconstruction of a spiny dendrite from **a**, showing various spine shapes (left), its embedding in dense neuropil (middle) and the 29 axons making a total of 39 putative synaptic connections at 38 spines (right). The scale cube refers to the center of the renderings. **d**, Distribution of spine lengths for the dendrite in **c**. **e**, Spine location (horizontal bars) and relative spine lengths (white portion of bars) along the dendrite (gold) with 3D renderings of example putative synaptic connections. Scale bars, 1 µm, referring to the center of the spine of interest. The overall width of the golden bar corresponds to the longest observed spine (3.96 µm). Proofreading of automated segmentation and 3D visualization and analysis as in **b**–**e** was applied to one dataset.

We now applied LIONESS to living hippocampal neuropil in the DG to unbiasedly visualize the architecture of this complex region. We reconstructed diverse cellular constituents, including myelinated and unmyelinated axons, spiny dendrites and glial cells (Fig. 3a,b, Supplementary Fig. 10a and Supplementary Video 3). Similar to EM, proofreading of automated segmentation remains a time-limiting factor, making it often preferable to selectively apply it to structures of interest. Reconstructing a 22-µm dendrite stretch revealed 38 spines of various morphologies (Fig. 3c), which is 1.7 spines per µm. Spine lengths ranged from 0.54 µm to 3.96 µm (1.77 µm ± 0.69 µm, mean ± s.d.) with unimodal distribution (Fig. 3d). We identified 29 axons where a bouton directly contacted a spine head, resulting in 39 potential synapses (Fig. 3c and Supplementary Fig. 10b). Most axons made single (20) or double (6) connections; however, triple and quadruple contacts were also observed. Both length and density quantifications are in keeping with previous data^38^. Figure 3e details spine length and position along the dendrite, together with example volumetric renderings.

### Molecularly informed reconstruction

**Fig. 4:**
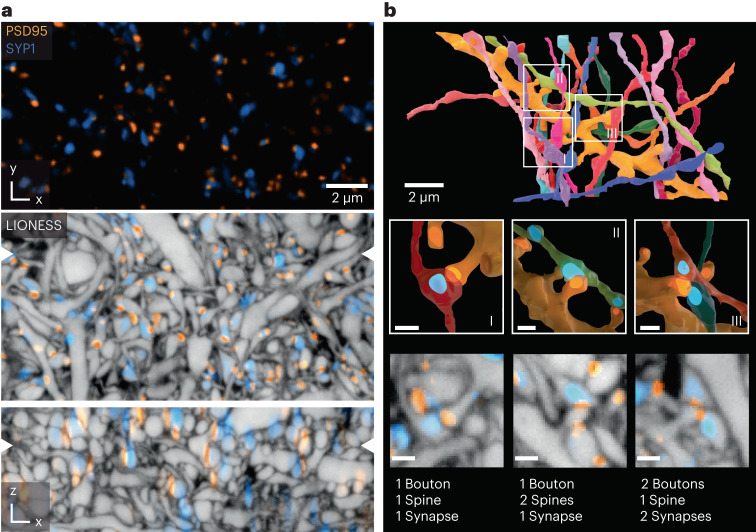
Molecularly informed reconstruction of living brain tissue. **a,** Confocal image of CA1 neuropil in organotypic hippocampal slice culture, with PSD95-HaloTag expressed via a genetic knock-in (*PSD95-HaloTag* mouse line), labeling all excitatory postsynapses (orange) and virus-assisted delivery of synaptophysin 1-eGFP (SYP1, blue), highlighting a subset of presynaptic terminals. Denoising (Noise2Void^51^) applied (top). Scale bar, 2 µm. Overlay with isotropically super-resolved volumetric LIONESS data (middle and bottom). Orthogonal planes in *xy* and *xz* directions represented as maximum intensity projections spanning 150 nm, with positions of corresponding planes indicated by arrowheads at image edges. Confocal SYP1 and PSD95 signals extend beyond the 3D super-resolved cellular structures defined by LIONESS. Data are representative for *n* = 4 biological replicates. **b,** 3D reconstruction of a selected dendrite (gold) from the same LIONESS volume with all synaptically connected axons as defined by postsynaptic presence of PSD95 (top). Magnified views as indicated by the boxes in the top panel, highlighting diverse geometric arrangements of synaptically connected boutons and spines together with renderings of PSD95 and SYP1 signals (middle). Renderings of molecule locations are based on thresholded confocal signals compressed in *z* direction to account for anisotropy of the confocal point-spread function. LIONESS planes from the corresponding subvolumes together with molecular information (bottom). Maximum intensity projections spanning 150 nm. Synaptic labeling and imaging together with LIONESS is representative of *n* = 4 biological replicates. Proofreading of automated segmentation and 3D visualizations were conducted for one dataset. Scale bars, 500 nm (middle and bottom). Scale bars refer to the center of the respective renderings.

**Extended Data Fig. 5:**
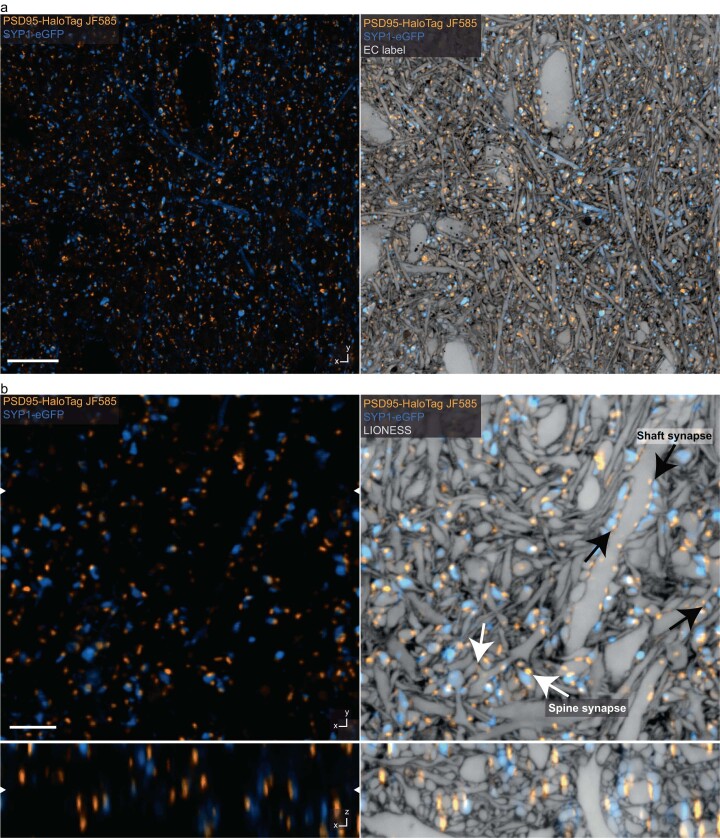
Structural and molecular information. **a**, Overview image of CA1 hippocampal neuropil in living organotypic slice culture from a transgenic mouse line expressing post-synaptic density protein 95 (PSD95)-HaloTag to label excitatory postsynapses (orange, STED, single plane, power distribution *z*-STED vs. *xy*-STED patterns: 80/20). A subset of presynaptic terminals were labeled with a synaptophysin 1 (SYP1)-eGFP fusion protein, encoded by a pseudotyped rabies virus (blue, confocal). *Left*: Molecular markers. HaloTag labeled with JF585. *Right*: Same region with structural context from additional extracellular labeling (STED, single plane, power distribution *z*-STED vs. *xy*-STED patterns: 80/20). Scale bar: 10 µm. **b**, Orthogonal planes in *xy*- and *xz*-direction of an imaging volume in CA1 from a different organotypic hippocampal slice sample. Labeling and color coding as in panel a. *Left*: Confocal imaging after denoising with Noise2Void. *Right*: Additional overlay with isotropically super-resolved LIONESS data, clarifying the relationship of molecularly defined entities (pre- and postsynapses) with cell- and tissue-structure. Diffraction-limited (confocal) synaptic molecule signals extend beyond corresponding structures recorded in LIONESS mode, particularly evident in the *xz*-view. White arrows indicate excitatory spine synapses, black arrows indicate excitatory shaft synapses. White arrowheads at image edges indicate the position of corresponding orthogonal planes (left image). The images are representative of n = 4 independent experiments. LIONESS images correspond to maximum intensity projections spanning 150 nm. Scale bar: 3 µm.

We next integrated key methods for live molecular labeling into tissue reconstruction. Affinity labels corroborated the identity of specific structures such as myelinated axons (Supplementary Fig. 11). Notably, light microscopy is unrivaled in visualizing specific proteins. As proximity between spines and boutons is a poor predictor of synaptic connectivity^6^, we complemented it with a molecular definition of synaptic sites. We used knock-in expression of HaloTag fused to endogenous PSD95 protein^39,40^, visualizing all excitatory postsynaptic terminals. For a proof-of-concept demonstration, we additionally applied adeno-associated virus (AAV) and pseudotyped rabies particles^41^ to express eGFP-coupled synaptophysin, visualizing a subset of presynaptic terminals (Fig. 4a, Extended Data Fig. 5 and Supplementary Video 4). LIONESS combined with confocal imaging of molecular markers in CA1 neuropil provided cellular context lacking with molecular readout alone. The punctate character of PSD95 signals allowed assigning them to specific spine heads or shafts in 99.9 % of cases (3,758 synapses; Supplementary Fig. 12), despite the lower resolution for the molecular labels. Combined structural/molecular information revealed various types of connections, including textbook-like single-bouton to single-spine contacts, two axons converging on a single spine and single boutons contacting two neighboring spines of the same dendrite with only one of them PSD95-positive (Fig. 4b). Overall, we identified 16 axons in molecularly verified synaptic contact with the reconstructed dendrite stretch, making 18 connections, while refuting one connection where morphology was suggestive but PSD95 was absent. Excitatory synapses are preferentially located at dendritic spines, but can also occur on shafts, particularly on aspiny interneurons. We used combined molecular/structural information to determine the fraction of excitatory synapses with shaft location, equaling 8.3% in Fig. 4 and 14.7% in Extended Data Fig. 5b. Comparison with confocal readout of synaptic molecules further illustrated augmented 3D definition with LIONESS (Fig. 4a and Extended Data Fig. 5b).

### Morphodynamics and activity

**Extended Data Fig. 6:**
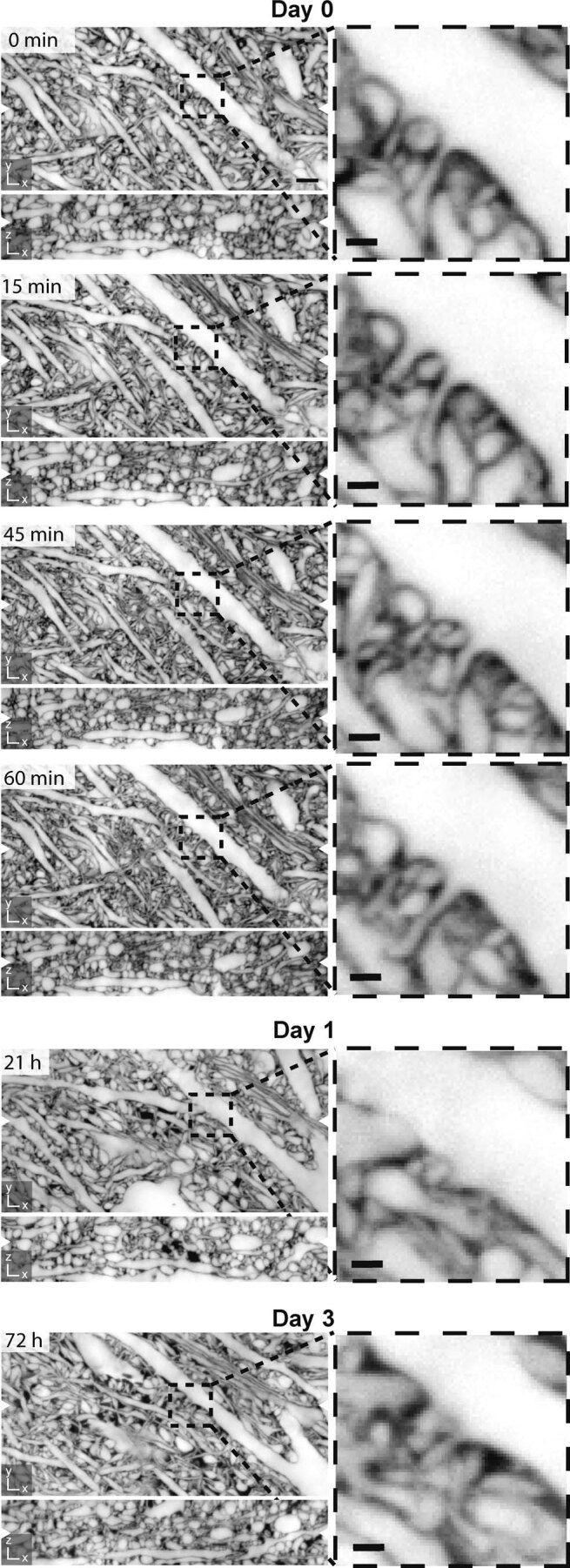
Structural dynamics in repeated volumetric LIONESS acquisition over 3 days. Corresponding orthogonal planes in *xy*- and *xz*-directions from 6 consecutive LIONESS measurements of the same volume in the neuropil of an organotypic hippocampal slice culture. The volume was initially imaged 4 times within one hour and then again after one day and after three days. Magnified views: Subregion with dendritic spines revealing morphodynamics. Scale bars, overview: 2 µm, magnified views: 500 nm. White arrowheads at image edges indicate the position of corresponding orthogonal planes. Maximum intensity projections spanning 150 nm. Additional dark regions on day 1 and day 3 likely represent branched processes of a damaged cell that took up dye after repeated manual mounting of the sample (supported by a membrane for interface tissue culture), transfer to the microscope, volumetric imaging, unmounting, and transfer back to the tissue culture incubator. The specific measurement sequence applied here was done for n = 1 sample.

**Extended Data Fig. 7:**
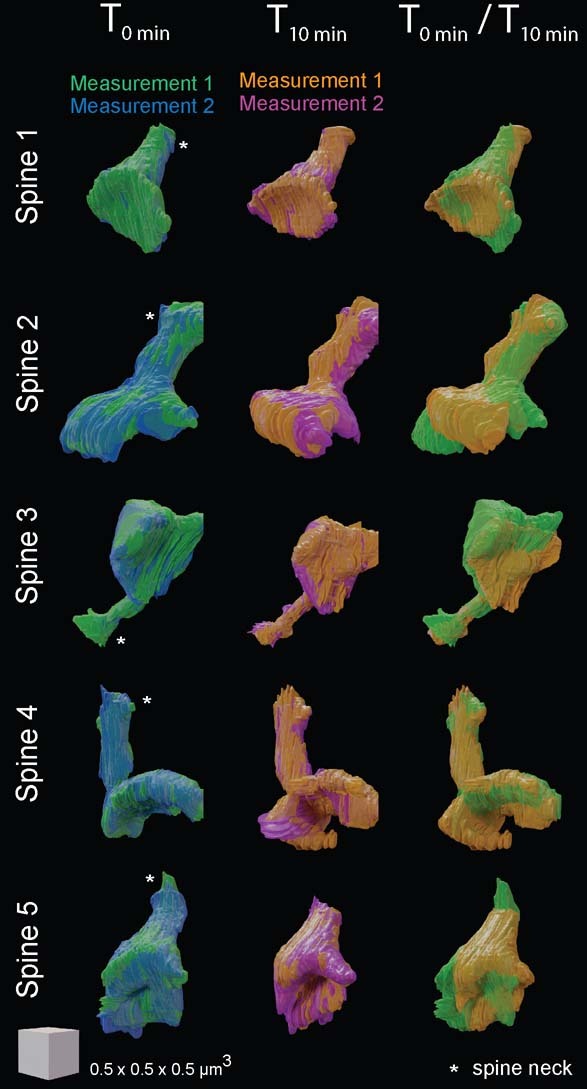
Image restoration and segmentation inaccuracy in relation to biological motion. Overlaid manual segmentations of individual dendritic spines from paired LIONESS measurements in neuropil of living organotypic hippocampal slice cultures recorded across n = 2 independent samples, excluding or allowing biological motion between measurements. We simultaneously generated two LIONESS measurements of the same imaging volume at time point T_0 min_ by dialing in 20 µs dwell time at each voxel and assigning photons from the first 10 µs to measurement 1 (green 3D reconstructions of individual spines) and those from the second 10 µs to measurement 2 (blue reconstructions of the same spines), such that biological motion was excluded for these two simultaneous measurements. Image restoration was applied to the raw data, selected spines were manually segmented and their 3D reconstructions were overlaid. Asterisks are placed near the attachment site of spine necks to the respective parent dendrites. We then repeated this dual measurement 10 minutes later at time point T_10 min_, again generating measurements 1 (orange reconstructions) and 2 (magenta reconstructions) for which biological motion was excluded. Finally, reconstructions corresponding to measurement 1 at the two different time points (T_0 min_/T_10 min_; green, orange) were overlaid by 3D-aligning spine necks to each other. Differences between the respective voxel exactly matched measurements 1 and 2 at single time points are attributable to measurement noise, inaccuracies in image restoration, and manual segmentation. Differences between the two time points (T_0 min_/T_10 min_) highlight changes in the biological structures. The scale cube refers to 500 nm in the center of the respective renderings.

Our low-exposure approach allowed repeated reconstruction of the same volume, revealing how subcellular morphologies evolved over time and pairing this with optical readout of activity. We first repeatedly imaged the same hippocampal neuropil volume over 3 d with LIONESS. This revealed morphological changes and movement of neuronal and non-neuronal subcellular structures in mutual context (Extended Data Fig. 6). As control, we tested at what level image restoration and segmentation inaccuracies limited detection of morphodynamics. We compared manual segmentations of the same dendritic spines from paired measurements where biological motion was either excluded in simultaneous duplicate measurement or, alternatively, where structural changes were possible during a 10-min measurement interval (Extended Data Fig. 7). Again, while uncertainties in restored 3D data and manual segmentation existed mostly at voxel level, more pronounced changes in spine shape were readily detectable.

**Extended Data Fig. 8:**
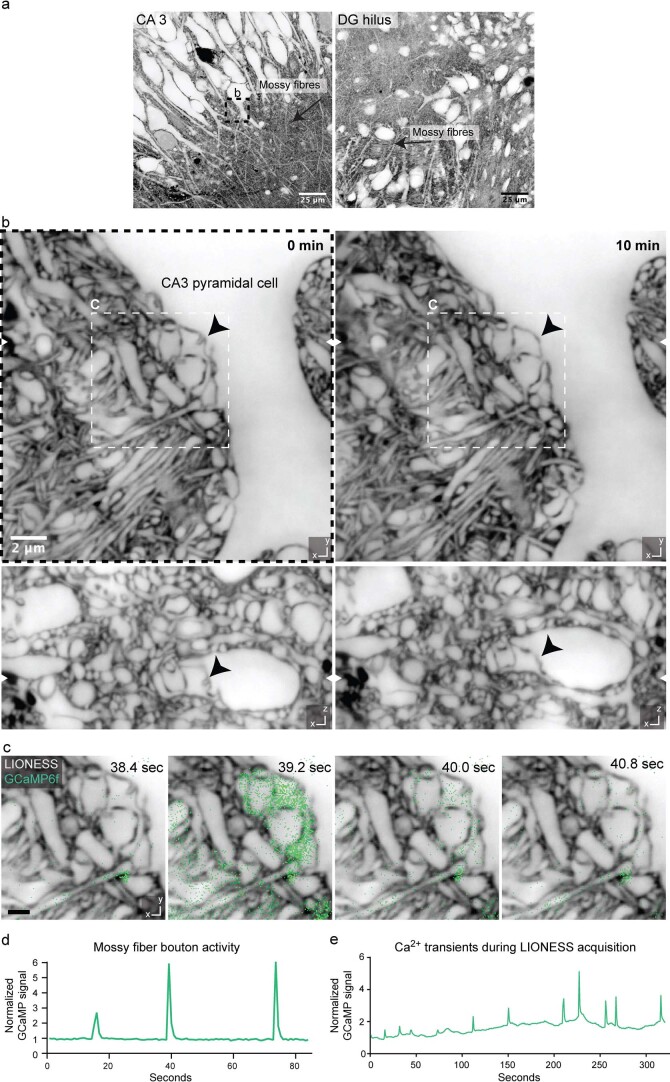
Correlating structure and morphodynamics with Ca^2+^-activity. **a**, Confocal overview images in organotypic hippocampal slice cultures from *Prox1-cre::Ai95* mice with mossy fibers conveying excitatory input from DG granule cells via the DG hilus (*right*) to CA3 pyramidal neurons (*left*). Scale bar: 25 µm. **b**, Isotropically super-resolved, volumetric LIONESS acquisitions in the stratum lucidum of CA3 at two timepoints (*left*: 0 minutes, *right*: 10 minutes) revealed morphodynamics of the complex interface between pre- and postsynaptic structures at mossy fiber to CA3 pyramidal neuron synapses. The black arrowhead marks a structure changing over time. The dashed frame in panel a indicates the position of the LIONESS volume. White arrowheads at image edges indicate the corresponding positions of *xy-* and *xz-*views. Scale bar: 2 µm. **c**, Plane from the LIONESS volume overlaid with diffraction-limited (confocal) signal from the calcium indicator GCaMP6f (green). LIONESS images are identical replicates providing structural context to the time-varying Ca^2+^-signals. Scale bar: 1 µm. The GABA_A_ antagonist gabazine was applied to increase activity. **d**, GCaMP signal of the conspicuous mossy fiber bouton shown in panel c as a function of time. Total signal from a rectangular region enclosing the mossy fiber bouton (roughly corresponding to the upper right quadrant of the image in panel c) was integrated and normalized to the first frame. **e**, GCaMP signal as a function of time (and position) recorded as an additional color channel during the volumetric LIONESS acquisition for timepoint 0 min in panel b. The images are representative of n = 4 technical replicates recorded from 3 biological specimens. LIONESS images are maximum intensity projections spanning 150 nm.

We then devised an all-optical approach to correlate 3D structure and signaling in a living cellular network. We focused on hippocampal circuitry, where mossy fibers originating from DG granule cells deliver excitatory input to proximal dendrites of pyramidal neurons in the CA3, forming boutons on complex spines (thorny excrescences)^42^ (Extended Data Fig. 8a,b). Using organotypic slices where all DG granule cells expressed the calcium indicator GCaMP6f (*Prox1-cre::Ai95*), we recorded calcium transients in individual mossy fiber boutons confocally, applying the GABA_A_ receptor antagonist gabazine to enhance network activity (Supplementary Video 5). LIONESS revealed the underlying cellular organization (Extended Data Fig. 8c,d and Supplementary Video 6). When repeating volumetric LIONESS imaging, mossy fiber boutons and their postsynaptic complex spines showed structural dynamics on the minutes timescale (Extended Data Fig. 8b). Signaling activity continued during LIONESS acquisition (Extended Data Fig. 8e).

**Fig. 5:**
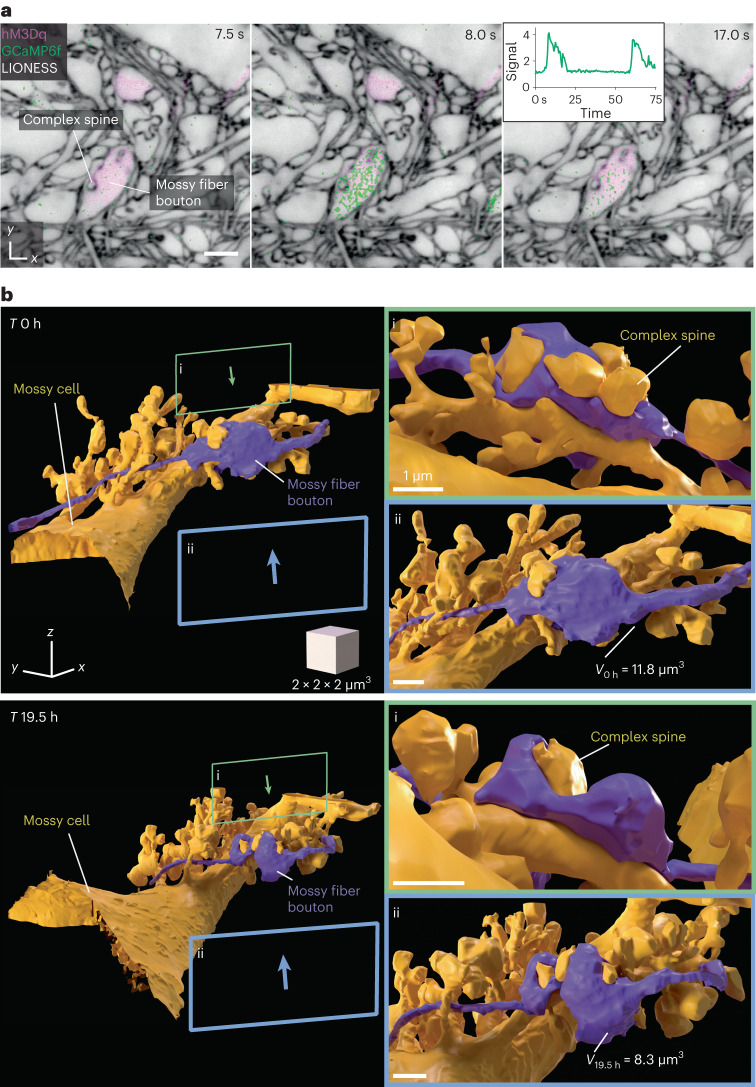
3D morphodynamics and chemogenetically induced Ca^2+^ activity in hippocampal mossy fiber-hilar mossy cell synapses. **a**, Single plane of an isotropically super-resolved LIONESS volume in the hilus of the DG in an organotypic hippocampal slice culture, where a subset of mossy fiber boutons expressed both the excitatory DREADD hM3Dq (together with cytosolic dTomato, confocal, purple, virally delivered) and the calcium indicator GCaMP6f (confocal, green, *Prox1-cre::Ai95* mouse). LIONESS and dTomato images are identical replicates, placing the overlaid time-varying Ca^2+^ signals after stimulation with the DREADD ligand CNO into structural context, showing three example points from a time series. Note that confocal signals extend beyond the structures defined by LIONESS. In particular, they partially originate from structures above and below the plane displayed here. Inset shows GCaMP signal (averaged pixel value normalized to first frame) as a function of time. LIONESS image is a maximum intensity projection spanning 150 nm. Scale bar, 2 µm. **b**, 3D reconstructions of a hM3Dq-expressing mossy fiber bouton (purple) and its postsynaptic partner, a hilar mossy cell (gold) with complex spines at two time points (top, day 0 (0 h); bottom, day 1 (19.5 h)). *V*_0h_ and *V*_19.5h_ are bouton volumes at the respective time points. Green (i) and blue (ii) frames indicate the viewing angles from opposite directions for the magnified views on the right. The structures designated by the lettering in **a** and **b** refer to the same bouton and complex spine. The scale cube refers to the center of the rendering. Scale bars in the magnified views correspond to 1 µm in the center of the respective bouton renderings.

We next developed a more refined approach for investigating activity and dynamics, combining chemogenetically targeted cell activation with Ca^2+^ imaging and dynamic reconstruction in the same living specimen. We expressed the virally encoded DREADD (designer receptor exclusively activated by designer drugs)^43^ hM3Dq sparsely in DG granule cells, enhancing neuronal excitation upon application of the bio-orthogonal drug clozapine-*N*-oxide (CNO). Together with transgenic GCaMP6f expression, this allowed controlling and imaging the activity of a large mossy fiber bouton in the DG hilus, before reconstructing it together with complex spines of the postsynaptic hilar mossy cell with LIONESS (Fig. 5a,b and Supplementary Video 7). Visualizing neighboring mossy fiber boutons further clarified spatial relationships (Supplementary Video 8). We investigated the structural evolution after 19.5 h (Fig. 5b), which revealed rearrangements in synaptic architecture and a bouton volume change from 11.8 µm^3^ to 8.3 µm^3^. These values are comparable to volumes of large mossy fiber boutons on CA3 pyramidal cells determined by serial sectioning EM in rat hippocampus^42^; however, with its applicability to living tissue, LIONESS has the capacity to repeatedly retrieve both activity and dynamic structural information directly in the living state, with the potential to follow structural plasticity and determine structure–function relationships.

### Electrophysiology

**Extended Data Fig. 9:**
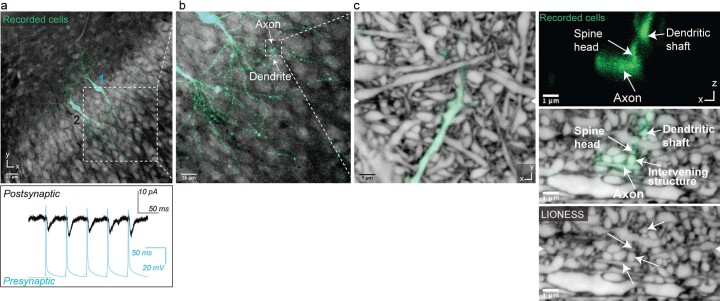
Correlating structural with electrophysiological information. **a**, Two CA1 pyramidal neurons after patch-clamp recording and filling with fluorescent dye in living organotypic hippocampal slice culture. Excitatory postsynaptic currents in neuron 2 (black trace in bottom panel, recorded in voltage-clamp configuration), elicited by action potentials in neuron 1 with short latency (<4 ms) (blue trace in bottom panel, triggered by current injection pulses in current-clamp configuration). Scale bar: 25 µm. Confocal image of positively labeled neurons (green) and extracellular label (gray) with low-numerical aperture objective. **b**, Region where axon of neuron 1 overlaps with a dendrite of neuron 2. Scale bar: 15 µm. **c**, Detailed view of overlap region with positively labeled structures (green) read out in confocal mode with a high-numerical aperture objective, embedded in the surrounding volume recorded with isotropically super-resolved LIONESS. Orthogonal views in *xy*- and *xz*-directions, with arrowheads at image edges indicating the position of the corresponding orthogonal sections. The images are representative of n = 3 experiments in different biological samples. Scale bars: 1 µm. Maximum intensity projections spanning 150 nm.

We reasoned that with LIONESS, light microscopy may not only be used to guide electrophysiology experiments, but to correlate electrical properties of single and synaptically connected neurons with the underlying neuronal architecture in the living state. We performed whole-cell patch-clamp recordings of two pyramidal neurons within the same specimen in CA1, as these often form synapses in organotypic culture^44^. To identify monosynaptically connected neurons, we elicited action potentials in one cell by current injection and measured responses in the other. We filled recorded neurons with fluorophores to re-identify the same cells after transfer to the super-resolution microscope. We first read out intracellular fluorophores confocally and then performed LIONESS (Extended Data Fig. 9). Zooming in on a putative contact, diffraction-limited readout indicated this as the site of electrophysiologically confirmed communication. Only comprehensive, 3D super-resolved delineation with LIONESS revealed the deception by disclosing an intervening, unlabeled neuronal process missed in confocal mode. This corroborated that LIONESS was suitable to multimodally retrieve and correlate structural with functional aspects of tissue architecture in living specimens and more powerful in doing so than diffraction-limited imaging.

### Bridging scales

**Extended Data Fig. 10:**
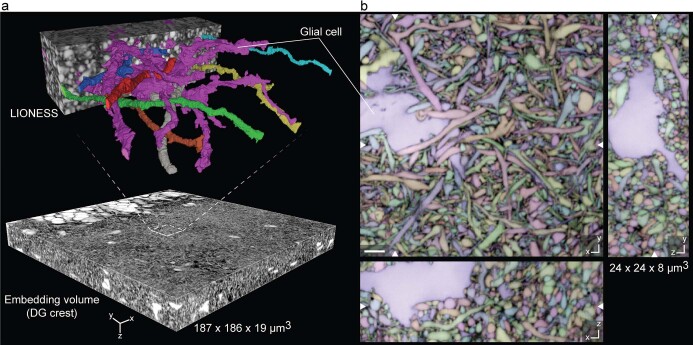
Meso-scale tissue context. **a**, Meso-scale overview volume acquired in confocal mode with select subvolume acquired and reconstructed using LIONESS in an organotypic hippocampal slice culture. A glial cell is 3D-rendered together with example neuronal processes. **b**, Three orthogonal planes from the automated segmentation of the LIONESS volume in panel a. Segmentation (color) and LIONESS data are overlaid. No proofreading was applied. White arrowheads at image edges indicate corresponding orthogonal planes. The same glial cell is indicated in both panels. Scale bar: 2 µm.

For extending analysis volumes and embedding into the meso-scale context, we followed two straightforward strategies. First, recording multiple partially overlapping volumes allowed them to be 3D registered, such that segmentation smoothly extended over the borders. We reconstructed a 70-μm-long stretch of mostly parallel axon fibers in acutely prepared alveus from four continuous imaging volumes, capturing ~3 mm cumulative axon length (Supplementary Fig. 13). Second, we embedded LIONESS into larger volumes at confocal resolution. Imaging a 650,000 µm^3^ volume in the DG crest gave positional context and allowed identification of larger objects such as cell somata and major dendritic branches, whereas LIONESS revealed neurites of DG granule cells and other neurites embedded in the invaginations of a glial cell (Extended Data Fig. 10 and Supplementary Videos 9 and 10). Imaging across spatial scales thus yields information on cell position and identity, extending the interpretation of morphodynamic and connectivity analysis by LIONESS.

## Discussion

We developed a technology to reconstruct living brain tissue and its time evolution, paired with molecular information and manipulation and readout of activity, thus bridging the gap between highly accurate but static EM representations and light microscopy reconstruction of positively labeled, incomplete subsets of cells.

Despite comprehensiveness of reconstruction unprecedented in light microscopy, there are limitations to address in future work. The detail unraveled at our $\lesssim$130-nm isotropic 3D resolution may be surprising when comparing to ~30-nm serial sectioning^6,10^ for EM connectomics or 8-nm voxel size in focused ion-beam scanning^3,45^ EM. With ECS labeling, a separating fluorophore layer was detectable also at a resolution lower than the thickness of this layer; however, the spine detection rate is currently considerably inferior to EM and the very finest structures cannot be unambiguously traced or assigned, especially when in highly complex arrangements such as thin tortuous axons in neuropil. Nevertheless, positive labeling or rabies circuit tracing^41,46,47^ may highlight select structures, while LIONESS provides nanoscale-resolved context. As a caveat, deep-learning image restoration does not a priori guarantee that biological structures are represented faithfully, requiring validation when adopting the technology. Predictions varied mostly at voxel level but restoration inaccuracies beyond that also factor into overall reported accuracy and the ability to distinguish biological motion from restoration errors. Both for image restoration and segmentation, we observed a certain model transferability between regions and datasets, also reflecting diversity in our training data. Nevertheless, with the high time cost for manual annotation, the amount of used training data is a limiting factor in automated segmentation. High-complexity regions, such as CA1 neuropil, required substantial human input to proofread automated segmentations, such that we focused on selected structures. Improving these various factors would be a prerequisite if this technology were to be employed for connectomics; however, our parameter search for image acquisition, processing and segmentation was in no way exhaustive, offering possibilities for future improvements and further reduced light exposure. Common to all super-resolution technologies, LIONESS poses high demands in imaging quality and optimum sample and imaging conditions were required for reconstruction. Using only standard tools for aberration compensation, penetration depth is currently limited to few tens of µm, which may be alleviated by adaptive optics^48^. Correlating LIONESS with measurements after fixation will be useful, providing further possibilities for molecular characterization and large-scale super-resolution imaging^49^, potentially benefiting from fixation-compatible extracellular labeling^50^.

In summary, LIONESS opens up the decoding of complex, dynamic tissue architecture in living mammalian brain and other organs and may ultimately challenge the way we think about the extent and significance of plasticity in the central nervous system.

## Methods

Procedures were performed in accordance with national law (BGBLA 114 and Directive 522), European Directive 2010/63/EU and institutional guidelines for animal experimentation. Experiments were performed on organotypic hippocampal slice cultures and acutely prepared hippocampus. This involves organ extraction after killing the animal, which does not require ethics approval. Research involving human H9 embryonic stem cells (line WAe009, https://hpscreg.eu/cell-line/WAe009-A) and cerebral organoids derived thereof was approved by the Ethics Committee at the Institute of Science and Technology Austria (ISTA Ethics Committee, approval date 9 June 2020).

### Animals

Animals were housed in groups of 3–4 animals under controlled laboratory conditions (12-h light–dark cycle with lights on at 7:00; 21 ± 1 °C; and 55 ± 10 % humidity) with food (pellets, 10 mm) and autoclaved water ad libitum. The animals were housed in commercially available individually ventilated cages made from polysulfone with a solid cage floor, dust-free bedding (woodchips) and nesting material. If not stated otherwise, we used wild-type C57BL/6J mice. All transgenic lines (Supplementary Table 1) used in this study have been previously characterized. For experiments involving Ca^2+^ imaging, we crossed *Ai95* (GCaMP6f)^52^ and *Prox1-cre*. For experiments with *PSD95-HaloTag* mice^39,40^, both homozygous and heterozygous animals were used. For all experiments, male and female mice were used interchangeably.

### Organotypic hippocampal slice cultures

Hippocampal slices were obtained from 5–7-day-old mice of either sex and cultured on cell culture inserts with porous membranes. Mouse pups were decapitated and the hippocampus was isolated while the brain was submerged in ice-cold sterile filtered HBSS without Ca^2+^ and Mg^2+^ (Gibco, 14175-053) supplemented with 10 mM glucose, using a stereo microscope. Hippocampi were cut into 350-µm thick slices and placed on round porous membranes with 4-mm diameter (PTFE membrane; Merck, FHLC01300) that were placed on cell culture inserts with a porous membrane (Millicell, PICM0RG50) for interface culture. The inserts with the slices were placed in dishes (Greiner, 627161) with 1 ml culture medium. We adapted the medium recipe during the course of experiments, as quality of cultures deteriorated with the same nominal composition. We found that 78.5% minimum essential medium (Gibco, 11095-080), 15% heat-inactivated horse serum (Gibco, 26050070), 2% B27 supplement (Gibco, 0080085SA), 2.5% 1 M HEPES (Sigma, M3375-100G), 1.5% 0.2 M GlutaMax supplement (Gibco, 35050-061) and 0.5% 0.05 M ascorbic acid (Sigma, A5960-25G), with additional 1 mM CaCl_2_ and 1 mM MgSO_4_ produced satisfactory results and incubated at 37 °C and 5% CO_2_. The medium was changed the day after preparation and then every 3–4 d.

### ECS labeling

For ECS labeling, artificial cerebrospinal fluid (ACSF) was prepared from a 10× stock solution with MgCl_2_ and CaCl_2_ added freshly before carbogen bubbling, whereas ascorbic acid and Trolox were added after bubbling. ACSF consisted of 125 mM NaCl, 2 mM CaCl_2_, 1.3 mM MgCl_2_, 4.8 mM KCl, 26 mM NaHCO_3_, 1.25 mM NaH_2_PO_4_, 7.5 mM HEPES (Gibco, 15630056), 20 mM d-glucose (Sigma, G8270-1kg), 1 mM Trolox (Sigma, 238813) and 1 mM ascorbic acid (Sigma, A5960-25G) at pH 7.4. Thereafter, fluorescent dye (Atto 643 (Atto-Tec, AD 643-25), SulfoAtto 643 or Abberior STAR 635 P (Abberior, ST635P)) was added from 5 mM stocks (dissolved in ACSF) to a final concentration of 150 µM. A 2-µl droplet of the dye-containing imaging solution was put on a no. 1.5H coverslip (Bartelt, 6.259995) that had been placed in an imaging chamber (RC-41, Warner Instruments). Using fine forceps, brain slices with the membrane attached were then carefully put onto the droplet, such that the slice was oriented toward the coverslip. A slice anchor gently kept the sample in place. Immediately afterwards, further imaging solution at room temperature was added. The imaging chamber was then placed onto the stage adaptor of the STED microscope (see below). The data in the manuscript were acquired using Atto 643 (except for Fig. 5 and Supplementary Fig. 8 (left panel) where SulfoAtto 643 was used).

### Acute preparation of whole hippocampus and labeling

Hippocampi were extracted from 5–7-day-old mice of either sex. Mouse pups were decapitated and the hippocampus isolated while the brain was submerged in ice-cold sterile filtered HBSS without Ca^2+^ and Mg^2+^ (Gibco, 14175-053) supplemented with 10 mM glucose, using a stereo microscope. The whole hippocampus was then submerged in freshly carbogenized ACSF with 150 µM Atto 643 dye and incubated for 10 min at room temperature with gentle agitation. Afterwards, entire hippocampi were placed on a no. 1.5H coverslip that had been placed in an imaging chamber (RC-41, Warner Instruments) with the alveus region facing the coverslip. A slice anchor gently kept the sample in place when freshly carbogenized ACSF with 150 µM Atto 643 dye was added for imaging. The imaging chamber was then placed onto the stage adaptor of the STED microscope.

### Generation of cerebral organoids

H9 human embryonic stem cells (https://hpscreg.eu/cell-line/WAe009-A) were obtained from a commercial provider (WA09, WiCell). Research involving human H9 embryonic stem cells and cerebral organoids derived thereof was approved by the Ethics Committee at the Institute of Science and Technology Austria (ISTA Ethics Committee, approval date 9 June 2020). Cells were dissociated to single cells using Accutase (Gibco). A total of 2,500 cells were transferred to each well of an ultra-low-binding 96-well plate (Corning) in mTeSR1 medium supplemented with 50 μM Y-27632 (STEMCELL Technologies). Cells were allowed to aggregate to embryoid bodies and fed every second day. On day 3, supplements were removed and from day 6 the generation of cerebral organoids was performed according to Lancaster and Knoblich^36^. Briefly, embryoid bodies were transferred to neural induction medium in low-adhesion 24-well plates (Corning) and fed every second day for 5 d until formation of neuroepithelial tissue (day 0 of cerebral organoid formation). Neuroepithelial tissue-displaying organoids were embedded in Matrigel droplets (Corning, 356234) and grown in cerebral organoid medium supplemented with B27 without vitamin A (Gibco) and fed every other day. After 4 d, tissues were transferred to cerebral organoid medium supplemented with B27 containing vitamin A and placed on a horizontal shaker. Cerebral organoids were fed twice a week.

### LIONESS imaging

STED microscopy was performed at room temperature on an inverted STED microscope (Abberior Instruments, Expert Line) with pulsed excitation and STED lasers. A 640-nm laser was used for excitation and a 775-nm laser for stimulated emission. A silicone oil immersion objective with 1.35 NA and a correction collar (Olympus, UPLSAPS 100XS) was used for image acquisition. The fluorescence signal was collected in a confocal arrangement with a pinhole size of 0.6 or 0.8 airy units. For detection a 685/70-nm bandpass filter (Chroma, F49-686) was used and a 50:50 beam splitter (Thorlabs, BSW29R) distributed the signal onto two photon-counting avalanche photodiodes, allowing for stronger excitation without saturating detectors. Both detection channels were added up using Fiji^53^ v.2.3.0/1.53f (Fiji/process/calculator plus/add), photon counts were inverted and data were saved in 16-bit TIFF format. The pulse repetition rate was 40 MHz and fluorescence detection was time-gated. LIONESS volumes were acquired with 10-μs pixel dwell time, 2.9-µW (640 nm) excitation laser power and 90-mW STED laser power. An SLM imprinted incoherently overlapped phase patterns for predominantly axial resolution increase (π-top-hat phase modulation, z-STED) and for predominantly improved fluorescence quenching outside the central minimum (4π-helical phase modulation, 4π(*xy*)-STED) onto the STED beam. The SLM was also used to perform alignment directly in the sample, ensuring that the intensity minima of the two STED patterns spatially coincided and to optionally adjust low-order Zernike polynomials for empirical aberration correction. The power ratio of *z*-STED/4π(*xy*)-STED was 80/20. The voxel size was 50 × 50 × 50 nm^3^ for all LIONESS images. The acquisition scan mode was typically *xzy*, with the *y* direction being the slowest scan axis, using galvanometric mirrors for lateral (*xy*) scanning and a sample piezo stage (Physik Instrumente (PI) KG, P-736.ZRO) for axial (*z*) scanning. Image acquisition and microscope control were performed with Imspector software v.14.0.3052 and v.16.3.13031. For samples with additional positive labels (HaloTag ligand JF585, Synaptophysin-eGFP, *Thy1*-*eGFP*, GCaMP6f, FluoroMyelin Green, AlexaFluor488 and dTomato), additional color channels with diffraction-limited resolution using a 488-nm or 560-nm laser with typically 10-μs dwell time and 1.1–3.9-μW (488 nm) and 2–2.6-µW (560 nm) excitation power were used for recordings in confocal mode. These signals were collected using photon-counting avalanche photodiodes with a 525/50-nm (Semrock, F37-516) and a 605/50-nm (Chroma, F49-605) bandpass filter for eGFP and JF585 detection, respectively. The 488-nm and 640-nm excitations were conducted simultaneously, for 560-nm excitation a second line step was used to avoid spectral bleed-through into the far-red channel. Voxel size was again 50 × 50 × 50 nm^3^ for all images with *xzy* scan mode. The power values refer to the power at the sample, measured with a slide powermeter head (Thorlabs, S170C).

### Repeated volumetric live imaging

For evaluation of tissue photo burden with LIONESS versus conventional high-exposure STED (Extended Data Fig. 2) a 70 × 70 µm confocal overview scan was performed in a region of the neuropil in the CA1 region of an organotypic hippocampal slice. Next, the central 5 × 5 × 2.5 µm^3^ volume was exposed to STED in 20 consecutive volumetric scans in the *xyz* scan mode with a 70-µs voxel dwell time for long-exposure STED and 10 µs for LIONESS datasets. Excitation and STED powers were identical and corresponded to the parameters used in LIONESS imaging, with 90 mW STED power at 80/20 distribution between phase patterns. At 10 min after the last volume was acquired, a second 70 × 70 µm confocal overview scan was performed on the same region and plane as in the initial measurement.

For long-term repeated imaging of the hippocampal neuropil (Extended Data Fig. 6), the sample was mounted and placed on the microscope as described in the section on LIONESS imaging. For the first four acquisitions within 1 h, the sample was kept in place, with the imaging medium (carbogenized ACSF with 150 µM Atto 643) exchanged after 30 min. After that, the sample was placed back onto a cell culture insert and into the tissue culture incubator at 37 °C and 5% CO_2_ until the next imaging session 1 d later. The same procedure was repeated for the last imaging time point after 3 d.

For long-term repeated imaging of chemogenetically activated mossy fiber boutons (Fig. 5), the sample was placed back after the first imaging session onto a cell culture insert and incubated at 37 °C and 5% CO_2_. The medium was changed after 45 min to wash out residual CNO and the sample was placed into the tissue culture incubator until the second imaging session on the next day.

### PSD95-HaloTag labeling

Organotypic hippocampal brain slices of *PSD95-HaloTag*^39,40,54^ mice were live labeled using Janelia Fluor (JF)585-HaloTag ligand (Janelia Research Campus). The fluorescent ligand was dissolved in anhydrous dimethylsulfoxide to a stock concentration of 500 µM, aliquoted and stored at −20 °C. Before imaging, the fluorescent ligand was added to the culture medium at a final concentration of 500 nM (1:1,000 dilution) and incubated for at least 45 min at 37 °C.

### Viral vector assembly and synaptophysin labeling

Preparation of AAV and RVdG_envA_-CVS-N2c vectors has previously been described^41,47^. Briefly, AAV2-CaMKIIa-TVA-2A-N2cG (Addgene, #172363) vectors were pseudotyped with the AAVdj capsid protein by co-transfection of HEK293T cells (ATCC, CRL-3216). Three days later, the cells were collected and lysed and the viral stock was purified using heparin-agarose affinity binding. RVdG_envA_-CVS-N2c-nl.eGFP-Syp.eGFP (Addgene, #172380) were rescued using HEK-GT cells and then amplified and pseudotyped using BHK-eT cells (HEK-GT and BHK-eT cells were previously generated and described^41^). Viral vectors were purified and concentrated from the supernatant using ultracentrifugation and resuspended in PBS.

For live labeling of synaptic vesicles, first AAV-CaMKIIa-TVA-2A-N2cG was added to organotypic hippocampal slice cultures at 7–10 d in vitro for dual expression of the TVA avian receptor and the rabies N2c glycoprotein (N2cG). After 14 d, envA-pseudotyped, G-deleted CVS-N2c rabies viral particles were added for expression of a synaptophysin-eGFP fusion protein and additional eGFP expression in the cell nucleus (RVdG_envA_-CVS-N2c-nl.eGFP-syp.eGFP). At 4–5 d after addition of the rabies vectors, eGFP expression was strong enough for imaging.

### Myelin labeling

Live labeling of myelin was performed using FluoroMyelin Green (Thermo Fisher Scientific, F34651). The dye was diluted 1:300 in culture medium for organotypic hippocampal slices and incubated with the sample at 37 °C for at least 30 min before imaging.

### Calcium imaging

Cultured organotypic hippocampal slices of *Prox1-cre::Ai95* (GCaMP6f)^52^ mice shown in Extended Data Fig. 8 were ECS-labeled for LIONESS imaging as described above. To reduce the level of inhibition, 10 µM GABA_A_ receptor antagonist gabazine was added to the imaging medium at the start of the imaging session. A region of interest was first repeatedly imaged via confocal scanning (488 nm excitation, 1.1 µW) of an individual plane with 50 × 50 nm^2^ pixel size and 5 µs pixel dwell time (frame rate ~1.25 Hz) to detect GCaMP signals. After recording, the enclosing volume was scanned in LIONESS mode. The GCaMP recording was overlaid with a corresponding plane of the volumetric LIONESS acquisition in Extended Data Fig. 8c. The same volume was imaged a second time 10 min after the first acquisition. The sample was kept in place in between the two recordings.

### Chemogenetic activation with calcium imaging

Chemically targeted activation with simultaneous calcium imaging of neurons was conducted using AAVs containing a Cre-dependent DREADD^55,56^ construct (AAV-DIO-CAG-hM3Dq-2A-dTomato; Addgene #202036) added to organotypic hippocampal slice cultures of *Prox1-cre::Ai95* (GCaMP6f) mice at 4–6 d in vitro. Each transduced cell expressed both cytoplasmic dTomato and the excitatory designer receptor hM3Dq. Concentrated viral stock (7 × 10^11^ genome copies per ml) was first diluted 1:10 in culture medium and subsequently 5 µl was carefully placed on top of each slice. Weak fluorescence was already detectable ~3 d after transfection and live imaging was performed from day 9 onwards after viral transduction. To activate the designer receptor, CNO was added (3 µM final concentration) to the imaging medium (fluorophore-containing ACSF). The GCaMP signal was recorded via confocal scanning (488 nm excitation, 3.9 µW) of an individual plane using a pixel size of 100 × 100 nm^2^ and dwell time of 20 µs, which resulted in a frame rate of ~2 Hz. The GCaMP recording together with the dTomato signal were overlaid with a corresponding plane of the LIONESS acquisition for representation. For the inset in Fig. 5a, a square region of interest around the CNO-activated mossy fiber bouton was defined; the GCaMP signal was averaged over this region and normalized to the value in the first frame.

### Electrophysiology

Organotypic slice cultures were submerged in ACSF containing 125 mM NaCl, 25 mM NaHCO_3_, 25 mM d-glucose, 2.5 mM KCl, 1.25 mM NaH_2_PO_4_, 2 mM CaCl_2_ and 1 mM MgCl_2_, with pH maintained at 7.3, equilibrated with a 95% O_2_/5% CO_2_ gas mixture at ~22 °C (room temperature). Glass micropipettes were pulled from thick-walled borosilicate glass (2 mm outer diameter and 1 mm internal diameter) and filled with intracellular solution containing 135 mM K-gluconate (Sigma, G4500), 20 mM KCl, 0.1 mM EGTA (Sigma, E0396), 2 mM MgCl_2_, 4 mM Na_2_ATP (Sigma, A3377), 0.3 mM GTP (Sigma, G8877) and 10 mM HEPES (Gibco, 15630056), with the addition of 20 µM AlexaFluor488 hydrazide (Invitrogen, A10436) and 0.2% (w/v) biocytin (Invitrogen, B1592) as required. Pipettes were positioned using two LN mini 25 micromanipulators (Luigs and Neumann) under visual control on a modified Olympus BX51 microscope equipped with a ×60 water-immersion objective (LUMPlan FI/IR, NA 0.90, Olympus, 2.05 mm working distance). Two neurons were simultaneously recorded in the whole-cell patch-clamp configuration, with signals acquired on a Multiclamp 700B amplifier (Molecular Devices), low-pass filtered at 6 kHz and digitized at 20 kHz with a Cambridge Electronic Design 1401 mkII AD/DA converter. Signals were acquired using Signal 6.0 software (CED). Action potential phenotypes were recorded on sequential current pulse injections (−100 to +400 pA) in the current-clamp configuration. Neurons were identified based on morphological and action potential phenotypes. In current-clamp recordings, pipette capacitance was 70% compensated.

Synaptic connectivity was assessed by sequential current injection into either recorded cell in the current-clamp configuration, while recording excitatory postsynaptic currents from the other in the voltage-clamp configuration. Presynaptic action potentials were elicited by five 1–2 nA current injection pulses for 2–3 ms at 20 Hz. Putative monosynaptic connections were identified by excitatory postsynaptic current generation (peak current > 2.5 × s.d. of baseline noise) in the postsynaptic cell with short latency (<4 ms) from the presynaptic action potential peak. Recordings were analyzed using Stimfit^57^ and MATLAB-based scripts.

After recording, neurons were resealed by forming an outside-out patch on pipette retraction, before immersion in solutions for live imaging.

### SulfoAtto 643 synthesis and characterization

In a 5-ml round-bottom flask equipped with a magnetic stir bar, Atto 643 NHS-ester (ATTO-TEC, AD 643-35; 5.0 mg, 5.23 μmol, 1.0 equiv.) was dissolved in a mixture of 700 μl *N*,*N*-dimethylformamide (Fisher Scientific, D/3846/17) and 300 μl dH_2_O. *N*,*N*-diisopropylethylamine (Carl Roth, 2474.1) (6.9 mg, 53.8 μmol, 9.3 μl, 10 equiv.) and taurine (Carl Roth, 4721.1) (3.4 mg, 26.8 μmol, 5.1 equiv.) were added successively and the reaction mixture was allowed to incubate under stirring for 60 min before it was quenched by the addition of 10 μl glacial acetic acid (Carl Roth, 6755.1). Semi-preparative reverse-phase high-pressure liquid chromatography was performed on an Agilent 1260 Infinity II LC System equipped with a Reprospher 100 C18 column (5 μm, 250 × 10 mm at 4 ml min^−1^ flow rate). Eluents A (0.1% trifluoroacetic acid (TCI, T0431) in dH_2_O) and B (0.1% trifluoroacetic acid in acetonitrile (Honeywell, 34851-2.5L)) were used. The gradient was from 10% B for 5 min → gradient to 90% B over 35 min → 90% B for 5 min with 4.0 ml min^−1^ flow. Peak detection and collection were performed at λ = 650 nm and provided 4.5 mg (4.7 μmol) of the desired product as a blue powder after lyophilization with 91% yield. Characterization was performed using high-pressure liquid chromatography–mass spectrometry (Supplementary Fig. 14) on an Agilent 1260 Infinity II LC System equipped with Agilent SB-C18 column (1.8 µm, 2.1 × 50 mm). Buffer A was 0.1% formic acid (Fisher Scientific, A117-50) in dH_2_O; buffer B was 0.1% formic acid in acetonitrile. The gradient was from 10% B for 0.5 min → gradient to 95% B over 5 min → 95% B for 0.5 min → gradient to 99% B over 1 min with 0.8 ml min^−1^ flow. Retention time *t*_R_ = 3.03 min. Low-resolution mass spectrometry: calculated, 943 Da; found, 943 Da. Excitation and emission spectra were recorded on a TECAN INFINITE M PLEX plate reader (λ_Ex_ = 580 ± 10 nm; λ_Em_ = 620–800 ± 20 nm; ten flashes; 40 µs integration time; λ_Ex_ = 300–660 ± 10 nm; λ_Em_ = 700 ± 20 nm; 10 flashes; 40 µs integration time) with 200 nM solutions of SulfoAtto 643 in PBS (Carl Roth, 9143.2) in Greiner black flat-bottom 96-well plates (Carl Roth, CEK8.1) (Supplementary Fig. 14).

### Restoration network training

Volumetric paired low-exposure, low-SNR training input data and high-exposure, high-SNR ‘ground truth’ data were recorded in a voxel-exact mode by collecting low-SNR data during the first 10 µs voxel dwell time and additional photons during the remaining 60 µs dwell time. High-SNR ground truth for network training were thus generated by adding up counts from the total 70 µs dwell time in Fiji v.2.3.0/1.53 f (Fiji/process/calculator plus/add). Other imaging parameters were as described in the section ‘LIONESS imaging’ (2.9 µW (640 nm) excitation laser power, 90 mW STED laser power with power ratio of *z*-STED/4π(*xy*)-STED of 80/20, voxel size 50 × 50 × 50 nm^3^). The 76 volume pairs of 12.5 × 12.5 × 5 µm^3^ each were used for training. Volumes were taken from organotypic hippocampal and cerebellar slice cultures and the alveus region of acutely dissected hippocampi. Network training (v.CSBDeep 0.6.1)^29^ parameters were as follows: 3D mode, 32 × 32 × 32 pixel patch size, 190 patches per volume, 150 steps per epoch, 150 epochs, batch size 32 and training data were loaded as 16-bit TIFF files. Software was installed from GitHub (https://github.com/CSBDeep/CSBDeep). A workstation with the following hardware components was used: Intel Xeon W ‘Skylake’ W-2145, 3.60 GHz processor, 128 GB RAM, NVIDA GeForce RTX 2080Ti graphics card.

### Denoising

To denoise confocal images recorded simultaneously with the LIONESS data in Fig. 4 and Extended Data Figs. 3 and 5b, Noise2void^51^ (v.0.2.1) was applied to individual channels with the following parameters: noise2void 3D mode, patch size 32 × 32 × 32 pixels, each patch augmented with rotations and axis mirroring, 150 training steps per epoch, 75 epochs (SYP1-eGFP) or 100 (PSD95-HaloTag), batch size 16 (SYP1-eGFP) or 32 (PSD95-HaloTag). Software was installed from GitHub (https://github.com/juglab/n2v). A workstation with the following hardware components was used: Intel Xeon W ‘Skylake’ W-2145, 3.60 GHz processor, 128 GB RAM, NVIDA GeForce RTX 2080Ti graphics card.

### Image processing for display

All used lookup tables were linear except for Fig. 2, Extended Data Fig. 1a–c and Supplementary Figs. 4 and 8, where a color calibration bar is provided. Threshold adjustments for display purposes were applied linearly and to the whole image. Line profiles (Fig. 1d, Extended Data Fig. 1c and Supplementary Fig. 2) were created using Fiji, line width was 2 pixels.

### Volume extension

For stitching of volumetric images, the Fiji 3D stitcher was used (Fiji/Plugins/deprecated/3D Stitching; linear blending, fusion α2.0).

### Tracing of axons

Tracing of axons was conducted using WebKnossos^58^ (v.22.05.1) installed on a local server. The experimenter who acquired the data selected a total of nine axons that expressed eGFP as ground truth from three different volumetric LIONESS datasets recorded in the neuropil of organotypic hippocampal slice cultures from *Thy1-eGFP* mice and placed one seed point in each. A blinded tracer with access to the LIONESS channel including the seed point but not to the eGFP channel traced the respective axons in both directions from the seed point. Quantification of tracing lengths and errors was conducted by the experimenter who acquired the data using WebKnossos.

### Manual segmentation and proofreading

Planes for manual segmentation were first upscaled fivefold without interpolation (plane depth was kept at the original 50-nm spacing). Segmentation itself was performed using VAST^59^ v.1.3.0 and v.1.4.0. Software was downloaded from https://lichtman.rc.fas.harvard.edu/vast/. For proofreading of automated segmentations data were visualized using Neuroglancer (https://github.com/google/neuroglancer) and corrected using VAST v.1.4.0 and v.1.4.1. Proofreading was performed by one person. In Fig. 4b, connections were further checked by a second person.

### Automated segmentation

We based our implementation of the automatic segmentation pipeline on the pytorch_connectomics^34,35^ framework. We used a U-Net architecture and trained the neural network to produce affinity maps, which were then processed by a watershed algorithm to obtain the final segmentations.

During training, the U-Net required volume data and the corresponding manual ground-truth segmentation. First, to adapt the input datasets to the framework requirements and maximize its performance, we applied a pre-processing step, converting the volume data to an eight-bit format and stretching the intensity to cover the whole intensity range. Then, the pre-processed volumes together with the corresponding ground-truth segmentations were passed into the U-Net. The three key parameters during training were the sample size, the number of training iterations and the data augmentation. Given the anisotropic step size (fivefold upsampling in the *xz* or *yz* plane for manual segmentation) of the input volume we noticed that using a sample size of 128 × 128 × 64, with the lowest number corresponding to the non-upsampled axis, substantially improved the performance of the neural network over smaller sizes. We increased the number of training iterations from the default 100,000 to 500,000, which further helped reduce segmentation errors. We found this number of iterations to be a reasonable compromise between training time and inference performance. Finally, we enabled all available data augmentation techniques.

During inference, we passed the pre-processed volume data into the U-Net and obtained the affinity map as an output. At the inference time, we used the same sample size used during training, with appropriate padding if the input volume was small and test-time augmentation via axis mirroring. The values in the final affinity map corresponded to the mean of the values obtained for each augmented case. The output affinity map was processed using the watershed algorithm to produce the labeled automatic segmentation. Our pipeline combined two different watershed implementations. First, we applied the image-based watershed method^60^ (https://github.com/zudi-lin/zwatershed) on each slice to compute fragment masks. These were then passed to a volume-based implementation (https://github.com/zudi-lin/waterz), which was applied on the affinity map, producing the final segmentation. We used watershed thresholds in the range 0.2–0.4 to minimize oversegmentation but to also avoid merges, which tend to be more tedious to fix during proofreading. The resulting segmentations contained spurious segments, which we reduced during a final postprocessing step by removing those that consisted of too few voxels (fewer than ten) or slices (fewer than two). This last step substantially facilitated later proofreading. The resulting segmentations were then analyzed visually, using Neuroglancer and quantitatively, using metrics such as segment size distribution and split ratio of ground-truth segments with respect to automatic segmentations.

We trained the U-Net on an eight-GPU (NVIDIA 3090s) node, using 32 CPUs and 128 GB RAM during the 500,000 iterations, which took 6 d. Inference time falls in the 10–40-min range, depending on the size of the input volume and can be performed on a more modest compute node. In our case, we used a two-GPU (NVIDIA 3090s) node using eight CPUs. The post-inference watershed and segmentation cleaning operations were performed on the inference node and took 10–20 min to complete.

### Visualization

The 3D visualizations were performed either using VAST^59^ v.1.4.0. (Figs. 1a and 2a, Extended Data Fig. 10a, Supplementary Figs. 5, 6a, 8 and 10 and Supplementary Video 8), Neuroglancer (Fig. 3b, Extended Data Figs. 4 and 10b and Supplementary Figs. 7 and 13), Blender v.2.93.4 (Figs. 1a, 3c, 4b and 5b, Extended Data Fig. 7 and Supplementary Figs. 6b and 10) or Neuromorph^61^ v.2.8 (Fig. 3e). Blender-generated visualizations were produced based on 3D meshes extracted from segmentations using marching cubes (as implemented in Scikit-Image, Python v.2.7 or v.3.7.12). These 3D meshes were first smoothed in Blender using a vertex-based smoothing operation that flattens angles of mesh vertices and finally the scene was rendered using Blender’s Cycles rendering engine. Supplementary videos were generated with iMovie. The schematics in the top row of Fig. 1a were created with Biorender.com.

### Dendrite abstraction

For representing dendrite synaptic connectivity in Fig. 3e, we developed a visual spine analysis approach inspired by Barrio^62^, a software for visual neighborhood analysis of nanoscale neuronal structures. We computed surface meshes for all axons and dendrites based on the segmented neuronal structures. Next, we used Neuromorph^61^ to compute spine lengths by specifying the base and tip of each spine and plotted spine positions and relative spine lengths according to position on the dendrite. Spine lengths were computed between the base and tip of each spine, following the spine’s central axis (skeleton). We abstracted the complex 3D morphology and connectivity of a dendrite from three to two dimensions to reduce visual clutter, while preserving relative spine positions and spine lengths. To do so, we mapped a dendrite’s 3D skeleton structure to a simplified, but topologically correct, two-dimensional representation. We preserved all relative distance relations within a dendrite (distances between spines) and encoded spine length at each spine location. Spine lengths were represented as bars, scaled relatively to the largest spine length of the dendrite.

### Differentiating restoration inaccuracies from biological movement

Paired measurements, where biological motion was either excluded or possible in Extended Data Fig. 7, were performed as follows. For each of the displayed dendritic spines, an imaging volume was acquired with 20 µs total voxel dwell time at time point *T*_0 min_. The first 10 µs were set aside for measurement one (*T*_0 min_) and the second 10 µs for measurement two (*T*_0 min_). Due to the interleaved character of the volume measurements, no morphological changes were possible but photon counts at individual voxels slightly differed due to counting statistics and noise. This was repeated at the same location after 10 min to create measurement one (*T*_10 min_) and measurement two (*T*_10 min_). After LIONESS image restoration of each individual dataset, morphology was compared by manually segmenting individual spines using VAST v.1.4.0 and overlaying the segmentations using Blender v.2.93.4. For comparing measurements at *T*_0 min_ and *T*_10 min_, spines were manually aligned in 3D, maximizing the overlap in the neck region.

### PSD95 localization relative to neuronal structures

To find and visualize the center of PSD95 confocal signals in Supplementary Fig. 12, the Laplacian of Gaussian detector of the TrackMate plugin^63^ v.7.6.0 for Fiji v.2.3.0/1.53f (Fiji/plugins/tracking/TrackMate) was used.

### Statistics and reproducibility

In all images, representative data from single experiments are shown. For LIONESS imaging and reconstruction, optimum sample and imaging conditions were required, such that lower quality measurements were discarded. This manuscript presents a technological development and no conclusions about the biological system are derived. Accordingly, experimental replicates were performed to demonstrate technical reproducibility rather than to describe any biological variability and no statistical methods were employed to predetermine sample size. Technical replicates typically involved several biological specimens, as indicated below. Statistical analysis and plotting of the data in Fig. 2b and Supplementary Fig. 8 were performed with Microsoft Excel for Mac (v.16.59) and GraphPad Prism (v.9.0.2).

LIONESS imaging of cerebral organoids as depicted in Fig. 1a and Supplementary Figs. 6 and 7 was additionally repeated on similar specimens twice (*n* = 3) and the LIONESS volume displayed in the figures was selected for reconstruction. The direct juxtaposition of STED and LIONESS for the same STED light parameters in Fig. 1b,c was performed in *n* = 3 technical replicates from two samples. The images in Fig. 2a stem from one dataset and the data in Fig. 2b correspond to *n* = 4 technical replicates (different LIONESS imaging volumes containing a positively labeled dendrite stretch). These were recorded from a total of three different biological specimens (three different organotypic brain slices), with the additional datasets displayed in Supplementary Fig. 8. The images in Fig. 2c–e are representative of tracing nine axons in a total of *n* = 3 biological replicates. LIONESS imaging in neuropil of organotypic hippocampal slice cultures as in Fig. 3, Extended Data Figs. 1 and 10 and Supplementary Figs. 1, 4 and 10 was repeated over 20 times. Proofreading of automated segmentation and 3D visualization and analysis in Fig. 3b–e and Supplementary Fig. 10 was applied to one dataset. LIONESS imaging paired with PSD95-HaloTag/SYP1-eGFP live labeling as in Fig. 4a,b and Extended Data Fig. 5 is representative of experiments in *n* = 4 different biological samples. Proofreading of automated reconstruction and 3D visualization in Fig. 4b was performed for one specimen. LIONESS imaging in DREADD-expressing samples in Fig. 5 was performed in two biological replicates and proofreading of the automated segmentation and 3D visualization were performed in one of these.

Measurements of point-spread functions on gold beads (Extended Data Fig. 1a,b) were performed for routine microscope alignment (*n* > 20) and the measurements of effective point-spread function on fluorescent beads in Extended Data Fig. 1c are representative of *n* = 2 repetitions. The direct comparison of performance in confocal and STED imaging with the indicated phase modulation patterns in Extended Data Fig. 1d is representative of *n* = 3 technical replicates recorded in two biological samples. Direct comparison of conventional STED light exposure and LIONESS in Extended Data Fig. 2 is representative of *n* = 4 experiments from two biological samples. Here, repeated exposure of the same region with conventional, high-photon-load STED (Extended Data Fig. 2a) was reproduced with performing *xy* scanning only, showing the same negative effect. In Extended Data Fig. 3a, we excluded one dataset (*n* = 1) from the image restoration training for testing, whereas 75 volumes were included in the training. Images in Extended Data Fig. 3b are taken from *n* = 5 technical replicates recorded across four biological specimens. Acute preparation of hippocampus and LIONESS imaging of the alveus region as shown in Extended Data Fig. 4 and Supplementary Fig. 13 was repeated in *n* = 4 samples and the respective segmentations and analyses were performed on the two examples selected for display. Repeated LIONESS imaging of the same sample volume at various timings was performed in more than four samples. Of these, datasets in Fig. 5 and Extended Data Figs. 2b, 6 and 8 were selected for the respective figures to demonstrate specific timing aspects. Imaging over 3 d in Extended Data Fig. 6 was conducted in one sample. The five spines segmented at two time points in Extended Data Fig. 7 were from *n* = 2 independent samples. The data on LIONESS combined with Ca^2+^ imaging in Extended Data Fig. 8 are representative of *n* = 4 technical replicates recorded across three different biological samples. The data on combining LIONESS with patch-clamp recordings in Extended Data Fig. 9 are representative of *n* = 3 biological replicates.

The images in Supplementary Fig. 1b are representative of a large number of measurements (*n* » 20), as we performed all our LIONESS imaging with these fluorophores. In contrast, we discarded fluorophores that either exhibited poor STED performance or entered cells after *n* = 2 experiments (Supplementary Fig. 1a). Comparison of single versus split detection (Supplementary Fig. 2) is representative for *n* = 3 technical replicates in the same specimen. We performed all experiments within the region of optimum imaging performance (~25 × 25 µm^2^ laterally and ~8–10 µm axially, up to a depth of ~50 µm). The visualization of performance outside this region in Supplementary Fig. 3a,b is representative of *n* = 2 technical replicates in the same sample. For testing voxel-based uncertainty measures in Supplementary Fig. 4b, we excluded one dataset (*n* = 1) from the image restoration training for testing, whereas 75 volumes were included in the training. Manual segmentation in the neuropil of an organotypic hippocampal slice culture (Supplementary Fig. 5) was conducted in *n* = 1 dataset. The data in Supplementary Fig. 9 represent the same dataset as Fig. 2a. Displayed examples for correctly identified and missed spines are representative of multiple occurrences of these cases in the *n* = 4 technical replicates of this measurement (Fig. 2a and Supplementary Fig. 8). FluoroMyelin imaging as shown in Supplementary Fig. 11 was performed in *n* = 3 brain slices from two mice and the assignment of synaptic proteins in Supplementary Fig. 12 contained 3,758 synapses recorded in *n* = 3 measurements from two specimens.

### Reporting summary

Further information on research design is available in the Nature Portfolio Reporting Summary linked to this article.

## Online content

Any methods, additional references, Nature Portfolio reporting summaries, source data, extended data, supplementary information, acknowledgements, peer review information; details of author contributions and competing interests; and statements of data and code availability are available at 10.1038/s41592-023-01936-6.

## Supplementary information

Supplementary Information Supplementary Table 1, Supplementary Figs. 1–14 and Supplementary References

Reporting Summary

Supplementary Video 1 LIONESS volume in living hippocampal alveus - one (*xy* view). Full LIONESS stack (*xy* view) in the alveus region of an acutely prepared mouse hippocampus, corresponding to the dataset in Extended Data Fig. 4. The displayed *xy* view spans 25 µm × 25 µm and the volume covers 7.7 µm axially. Step size, 50 nm (154 optical sections).

Supplementary Video 2 LIONESS volume in living hippocampal alveus - two (*yz* view). Full LIONESS stack (*yz* view) in the alveus region of an acutely prepared mouse hippocampus, corresponding to the dataset in Extended Data Fig. 4. The displayed *yz* view covers 25 µm × 7.7 µm and the volume covers 25 µm in the third dimension. Step size, 50 nm (500 optical sections).

Supplementary Video 3 LIONESS volume in living hippocampal neuropil. Full LIONESS stack (x*y* view, followed by *xz* view) of a volume of DG in organotypic hippocampal slice culture, corresponding to the dataset in Fig. 3. Volume edge lengths are 23.2 µm × 22 µm × 6 µm. Step size, 50 nm.

Supplementary Video 4 Molecularly informed LIONESS volume in living hippocampal neuropil. Full LIONESS stack (*xy* view, edge lengths, 18 µm × 18 µm) of the entire PSD95 (orange) and synaptophysin (blue) labeled dataset shown cropped in Fig. 4. Step size, 50 nm (120 optical sections).

Supplementary Video 5 GCaMP recording, overview. Confocal recording of calcium transients using *Prox1-cre::Ai95* mouse organotypic hippocampal slice cultures expressing GCaMP6f, extracellularly labeled with Atto 643. The time series corresponds to a single plane of a region in the DG during application of the GABA_A_ receptor antagonist gabazine. Note that the sample was slightly mechanically shifted when gabazine was manually added. Acquisition frame rate was 1.25 Hz. Edge lengths, 70 µm × 70 µm.

Supplementary Video 6 GCaMP recording, synaptic bouton level. Full time series of the GCaMP6f recording after gabazine application in *Prox1-cre::Ai95* mouse organotypic hippocampal slice cultures shown in Extended Data Fig. 8c. LIONESS images are identical replicates providing structural context to the time-varying Ca^2+^ signals (confocal, green). Acquisition frame rate of the GCaMP signal was 1.25 Hz. LIONESS images are maximum intensity projections spanning 150 nm. Edge lengths, 7.5 µm × 7.5 µm.

Supplementary Video 7 GCaMP recording, chemogenetically activated single bouton. Full series of the GCaMP6f recording shown in Fig. 5a. Ca^2+^ transients of a mossy fiber bouton attached to a hilar mossy cell in *Prox1-cre::Ai95* mouse organotypic hippocampal slice cultures are visible. LIONESS (greyscale) and dTomato (confocal, orange, coexpressed with the DREADD hM3Dq) images are identical replicates placing the overlaid time-varying Ca^2+^ signals (confocal, green) after stimulation with the DREADD ligand CNO into structural context. Acquisition frame rate of the GCaMP signal was 2 Hz. LIONESS images are maximum intensity projections spanning 150 nm. Edge lengths, 14.8 µm × 14.4 µm.

Supplementary Video 8 3D reconstruction of mossy fiber bouton on hilar mossy cell, including neighboring boutons. Same reconstruction as in Fig. 5b on day 0, but including additional, hM3Dq-negative mossy fiber boutons from automated segmentation (left), and the thorny excrescences alone (right). Additional boutons are not proofread. Imaging volume was 15 × 15 × 8.5 µm^3^.

Supplementary Video 9 LIONESS volume in living hippocampal DG - one (*xy* view). Full LIONESS stack (*xy* view, edge lengths, 24 µm × 24 µm) of a volume of DG in organotypic hippocampal slice culture, corresponding to the dataset in Extended Data Fig. 10. Step size, 50 nm (160 optical sections).

Supplementary Video 10 LIONESS volume in living hippocampal DG - two (*xz* view). Full LIONESS stack (*xz* view, edge lengths, 24 µm × 8 µm) of a volume of DG in organotypic hippocampal slice culture, corresponding to the dataset in Extended Data Fig. 10. Step size, 50 nm (480 optical sections).

Supplementary Software Zip file containing custom software/code.

## Data Availability

Imaging data and models are available at the Institute of Science and Technology Austria’s data repository with 10.15479/AT:ISTA:12817 (https://research-explorer.ista.ac.at/record/12817).

## Code availability

Code related to this publication is available as Supplementary Software as a zip file and at https://github.com/danzllab/LIONESS under the MIT license.
