# Supplementary material for: Dense 4D nanoscale reconstruction of living brain tissue
Source: Nat Methods. 2023 Jul 10;20(8):1256–65. doi: 10.1038/s41592-023-01936-6 (PMC10406607; doi:10.1038/s41592-023-01936-6)
Supplement: Supplementary file 1 — Supplementary Table 1, Supplementary Figs. 1–14 and Supplementary References [file 41592_2023_1936_MOESM1_ESM.pdf]

nature methods

Article

https://doi.org/10.1038/s41592-023-01936-6

Dense 4D nanoscale reconstruction of living brain tissue

In the format provided by the authors and unedited

Transgenic line	Full name	Source (deposited by)	Cat#
Ai95	B6;129S-Gt(ROSA)26Sortm95.1(CAG-GCaMP6f)Hze/J	Jackson labs (H. Zeng)	024105
Prox1-cre	Tg(Prox1-cre)SJ32Gsat/Mmucd	MMRRC (N. Heintz)	036644-UCD
PSD95-HaloTag	PSD95-HaloTag	Seth G. N. Grant, Edinburgh	-
Thy1-eGFP [1]	STOCK Tg(Thy1-eGFP)MJrs/J	Jackson labs (J. Sanes)	007788

Supplementary Table 1

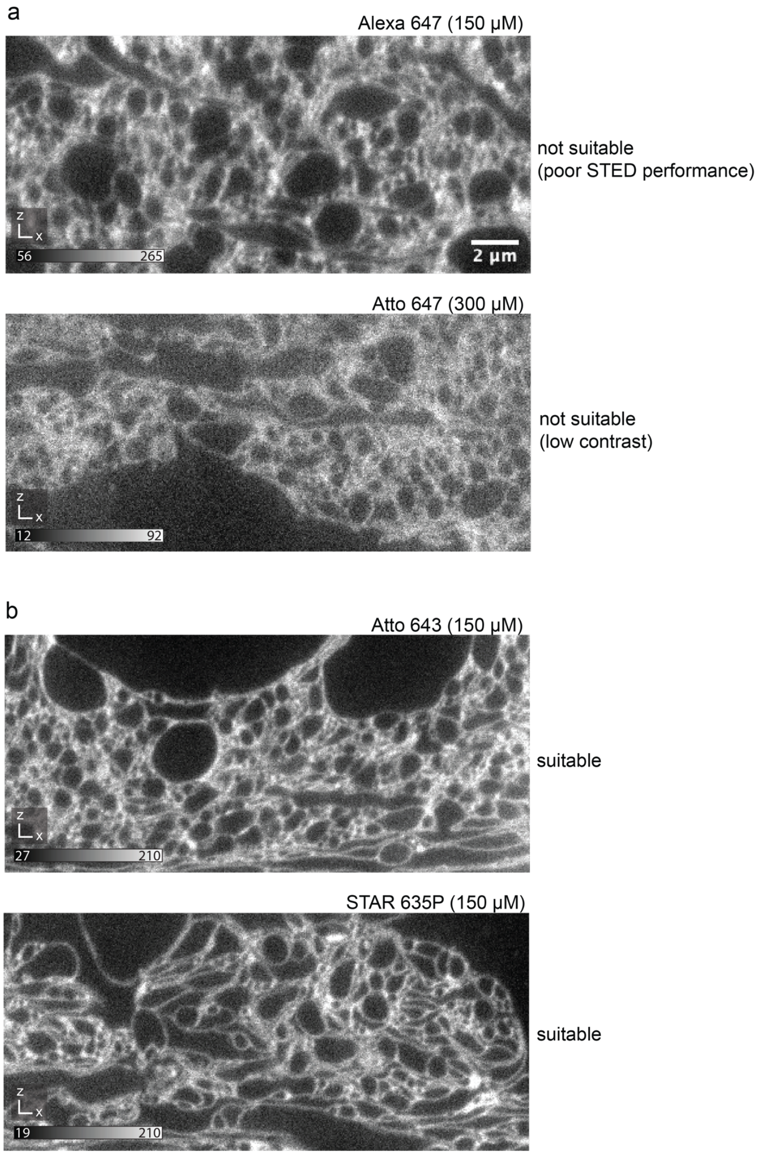

Supplementary Fig. 1| Fluorophore screening. **a**, Two examples of fluorophores yielding insufficient delineation of fine cellular structures, due to suboptimal STED performance (*top*) or poor extra- vs. intracellular contrast (*bottom*). **b**, Two examples of fluorophores with high STED performance and high extra- vs. intracellular contrast (Atto 643, Abberior STAR 635P), yielding adequate delineation of fine cellular structures. All images show raw *xz*-planes recorded with tissue-optimized STED patterns at isotropic resolution (π-top-hat plus 4π helical phase modulation receiving 80% vs. 20% of total power, respectively). Fluorophores applied at indicated concentrations to ECS in organotypic hippocampal slice cultures. The custom synthesized sulfonated variant of Atto 643 (SulfoAtto 643) was equally suited and used interchangeably with Atto 643. The experiments with suitable dyes are representative of n>20 repetitions. Dyes that exhibited poor STED performance or entered cells were discarded after n=2 experiments. Scale bar: 2 µm, valid for all images. Numbers in greyscale bars refer to raw photon counts.

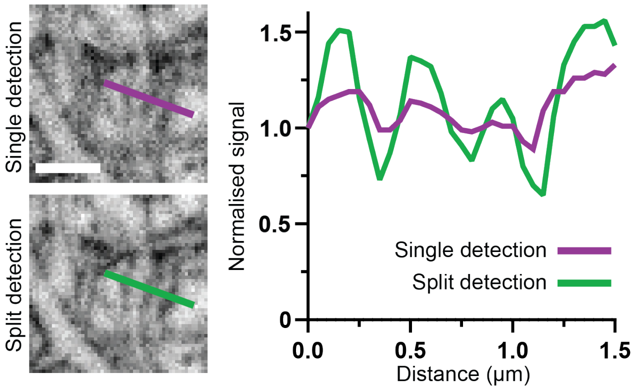

Supplementary Fig. 2| Detector dynamic range. Same region in organotypic hippocampal slice culture imaged with a single detector (*top*) or with a split detection path and two single-photon counting avalanche photodiodes as detectors (*bottom*). Line profiles over corresponding structures for single (purple) and split (green) detection, normalized to first data point. STED power and pixel dwell times were identical, and STED patterns for isotropic resolution (4π-helical and π-top-hat phase modulation) were used. Excitation power was doubled in the measurement with split detection, while operating the detectors in the linear detection regime. Single imaging planes in organotypic hippocampal slice cultures. The images are representative of n=3 technical replicates in the same specimen. Scale bar: 1 μm.

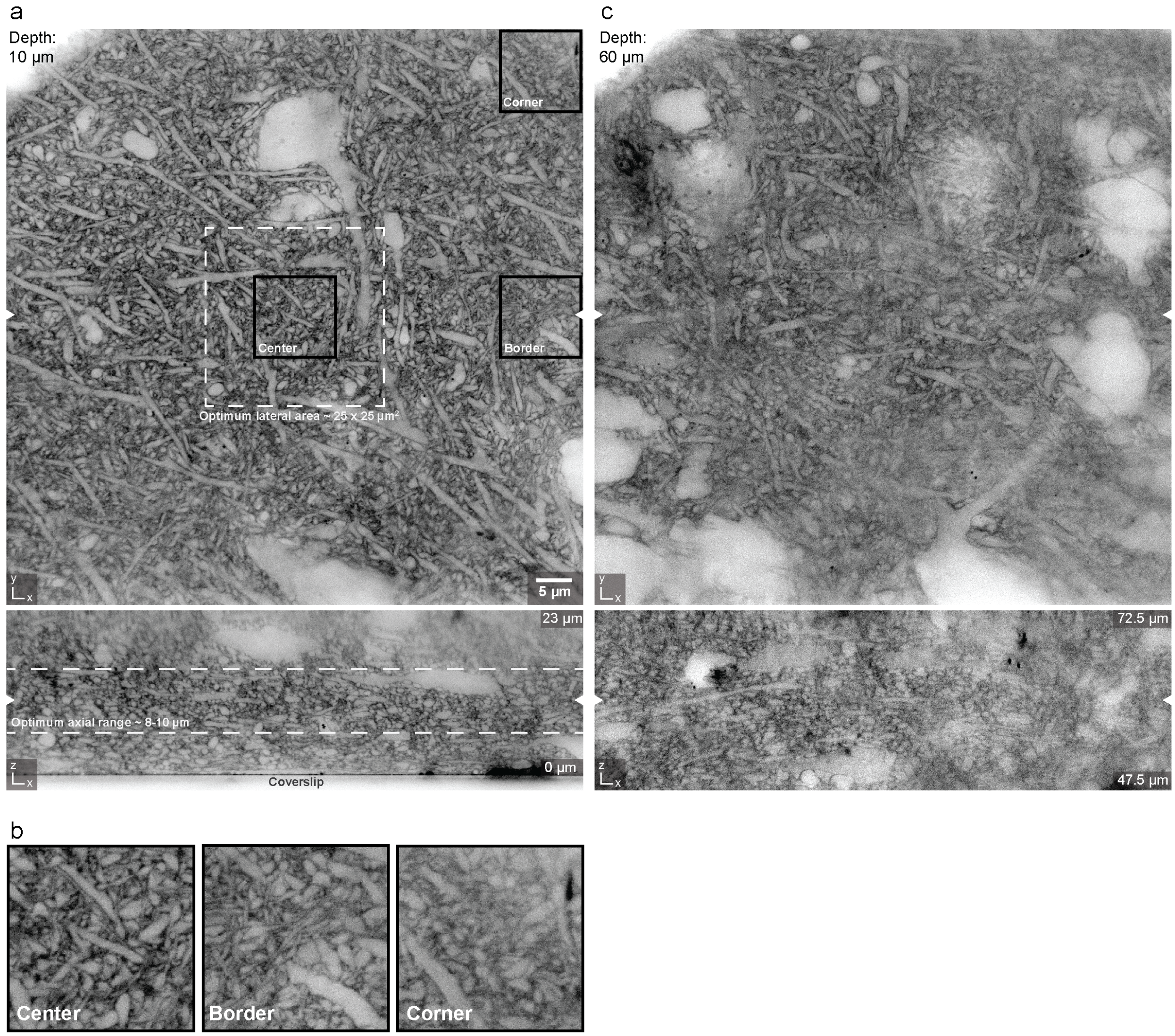

Supplementary Fig. 3| Imaging performance as a function of position. a, STED images recorded in *xy*- and *xz*-directions in neuropil of organotypic hippocampal slice culture, recorded around a central depth of 10 µm in the tissue. White arrowheads at image edges indicate position of corresponding orthogonal planes. Lateral and axial scan ranges were chosen to include also regions outside the central volume of optimum imaging quality. The region of optimum performance usually used in our experiments corresponded laterally approximately to the central 25 x 25 μm^2 (*top*, dashed white box). Axially, the optimum imaging range covers ~8-10 µm around the depth of optimum adjustment for spherical aberrations with the objective's correction collar and, optionally, the SLM (*bottom*, dashed white lines). Numbers in the right corners of the *xz*-view indicate depth below the coverslip/tissue surface. STED light patterns, power, and power distribution were the same as in LIONESS imaging, voxel dwell time was 70 µs. Single orthogonal planes were recorded. **b,** Magnified views of the regions indicated by black boxes in panel a. **c,** Same measurement around a central imaging depth of 60 µm with correction collar adjusted to that depth. The images are representative of n=2 experiments.

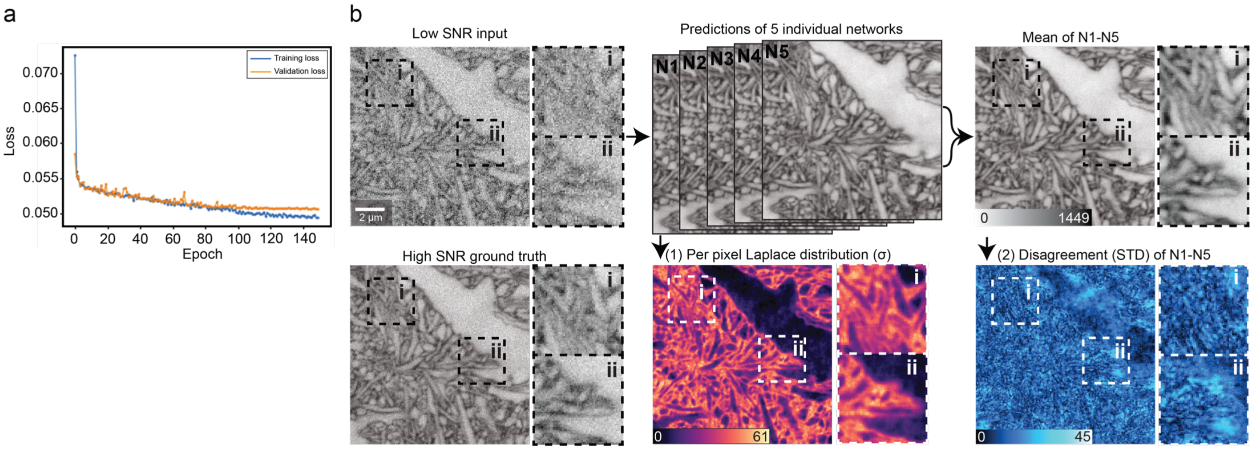

Supplementary Fig. 4| Image restoration training and validation. a, Training and validation loss as a function of epoch number of the image restoring deep artificial network. **b**, Validation of artificial network predictions on n=1 paired low- and high-SNR dataset that was not part of the network training, recorded with tissue-optimized STED in extracellularly labelled organotypic hippocampal slice cultures. Multiple regions of the volumetric data were examined. (1) Intrinsic probabilistic estimation of uncertainty (per pixel Laplace distribution scale parameter (σ)) for individual predictions (lower panel, *middle*) and (2) standard deviation of the mean (disagreement) of 5 trained networks N1-N5 for each voxel (lower panel, *right*). Raw data and network predictions are maximum intensity projections spanning 150 nm. Scale bar: 2 µm.

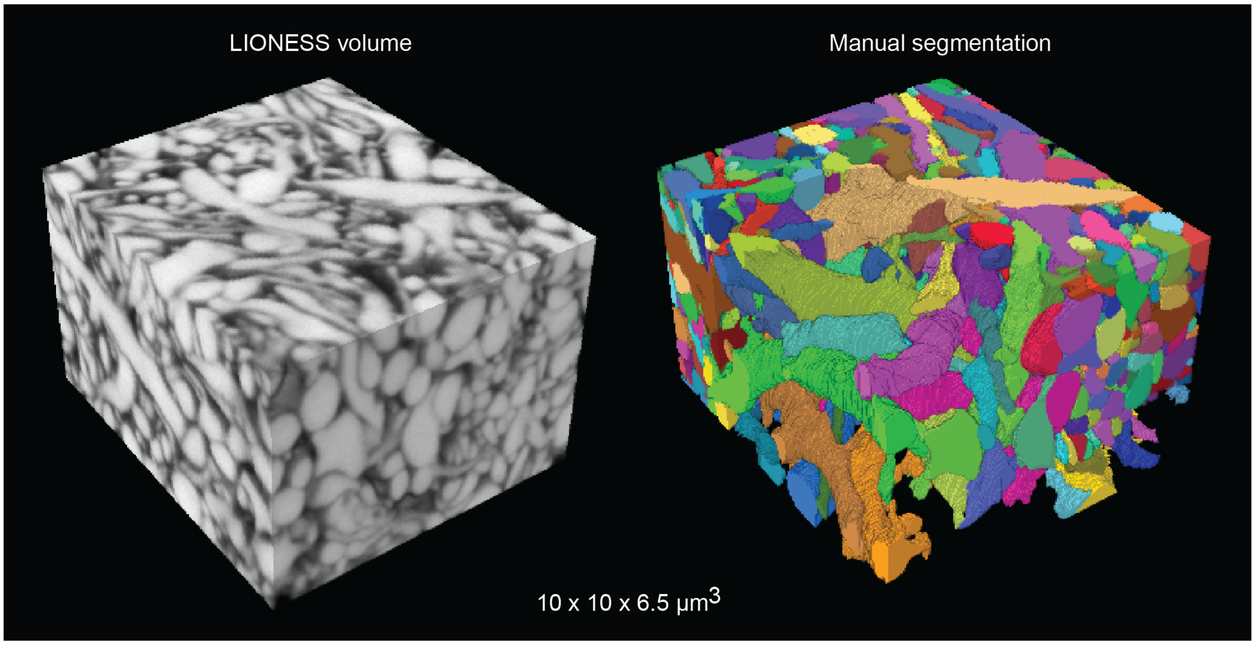

Supplementary Fig. 5| Manual segmentation. *Left*: LIONESS tissue volume in neuropil of organotypic hippocampal slice culture. *Right*: Manual segmentation using VAST Lite 1.3.0 and 1.4.0. The region in the foreground shows a partial segmentation. Manual segmentation was done on one dataset.

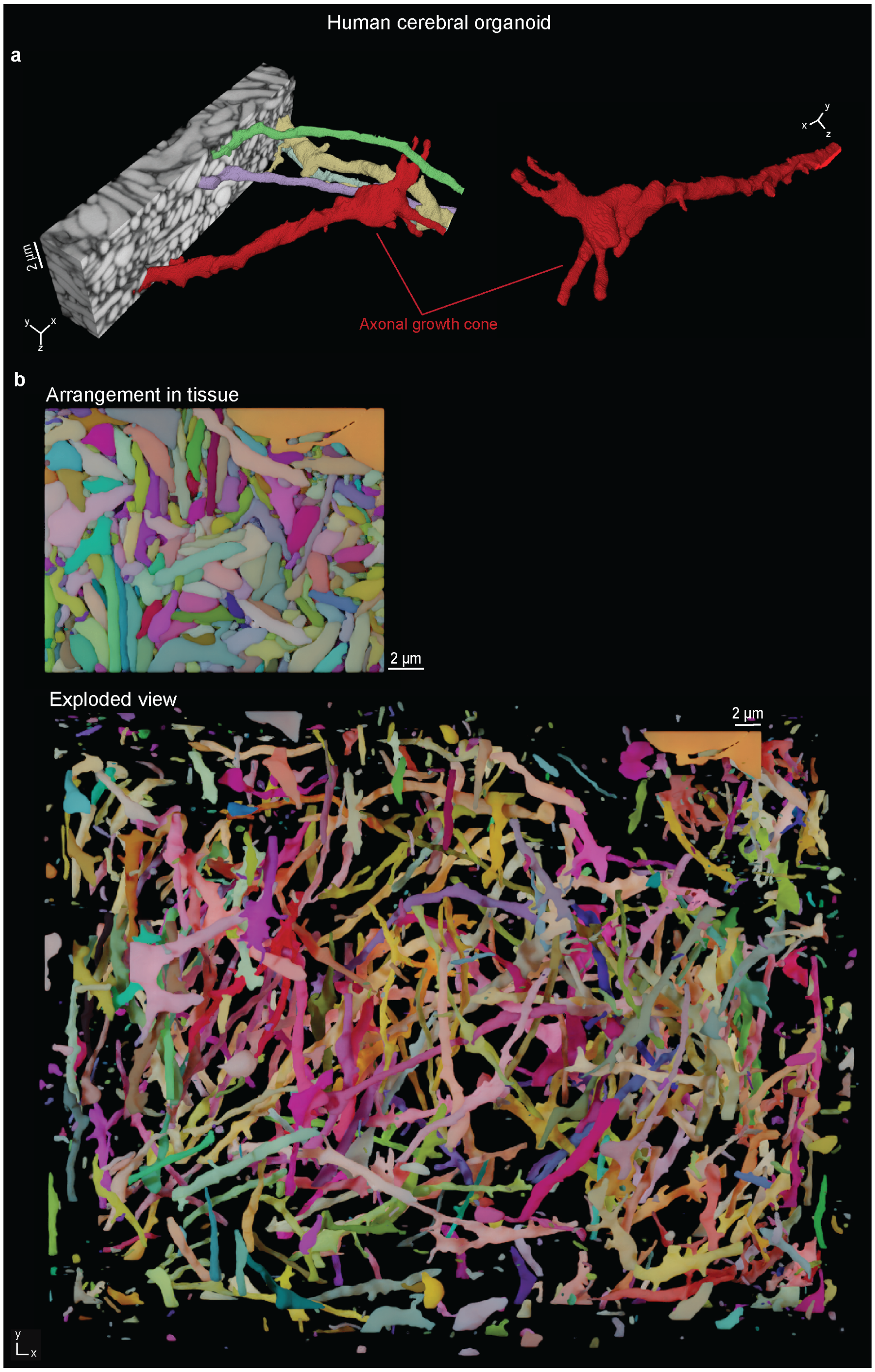

Supplementary Fig. 6| Reconstruction of living human cerebral organoid. a, *Left*: LIONESS volume of the human cerebral organoid in Fig. 1, eroded to reveal an axonal growth cone transmigrating the dense tissue and a selection of the structures it interacts with. *Right*: The same growth cone viewed from a different angle. **b**, Top view of the comprehensive organoid reconstruction in Fig. 1. (*top*), and exploded view of the same reconstruction (*bottom*). LIONESS imaging was performed in n=3 organoid specimens and one volume from those was reconstructed as shown here. Scale bar: 2 µm.

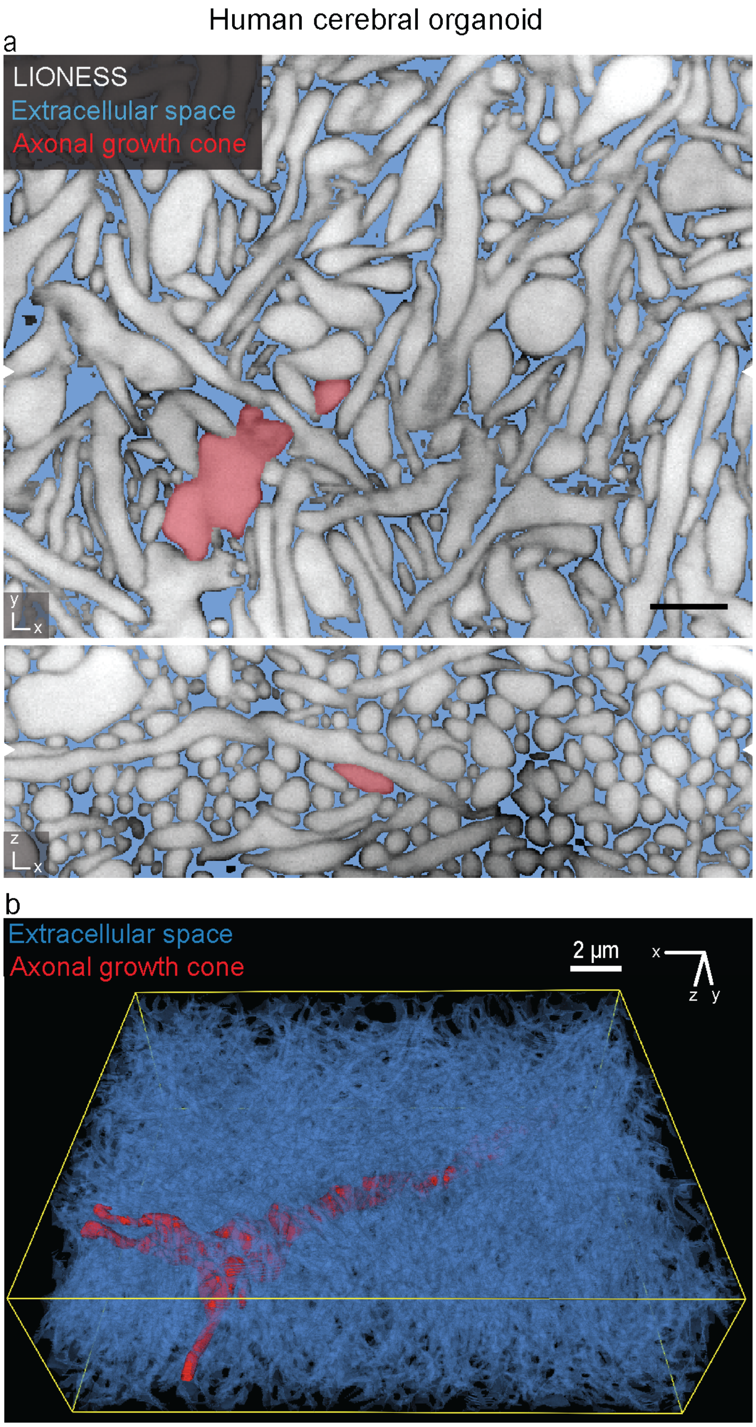

Supplementary Fig. 7| 3D-segmentation of extracellular space in a human cerebral organoid. a, Orthogonal views from the human cerebral organoid dataset in Fig. 1. Extracellular space is highlighted in blue, LIONESS data is shown in grey, and the same axonal growth cone as in Supplementary Fig. 6 is indicated in red. White arrowheads at image edges indicate corresponding orthogonal planes. Extracellular space was obtained as the space not occupied by cellular segments. Scale bar: 2 µm. **b**, 3D-reconstruction of the extracellular space (blue) with the axonal growth cone (red).

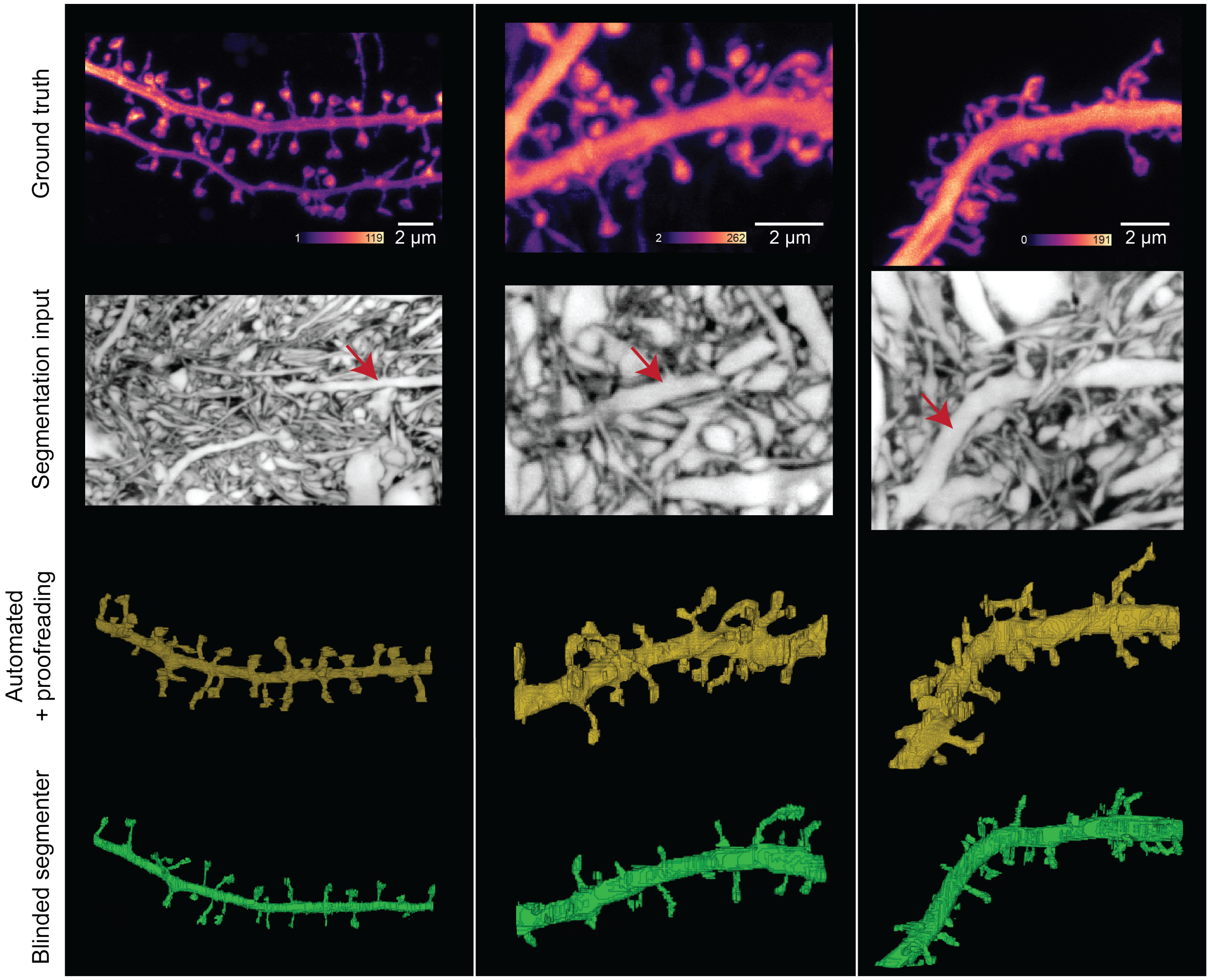

Supplementary Fig. 8| Validation of LIONESS segmentation. *Top row*: Maximum intensity projections of confocal imaging stacks with positively labeled dendrites from neuropil in n=3 different samples of organotypic hippocampal slice cultures generated from *Thy1-eGFP* mice, serving as sparse ground truth for LIONESS segmentations, together with the data in Fig. 2a forming the basis for the analysis in Fig. 2b. Scale bars: 2 µm. Calibration bars represent raw photon counts. *Second from top*: Isotropically super-resolved volumetric LIONESS acquisitions used as source data for segmentation. Red arrows indicate the dendrites corresponding to the positively labelled structure above. The images are representative for each of the three datasets. *Third from top*: 3D-reconstructions of LIONESS data with automated segmentation and additional proofreading by the experimenter who recorded the data (i.e. non-blinded to the eGFP channel). *Bottom*: Fully manual spine detection from LIONESS data by a segmenter blinded to the eGFP-channel.

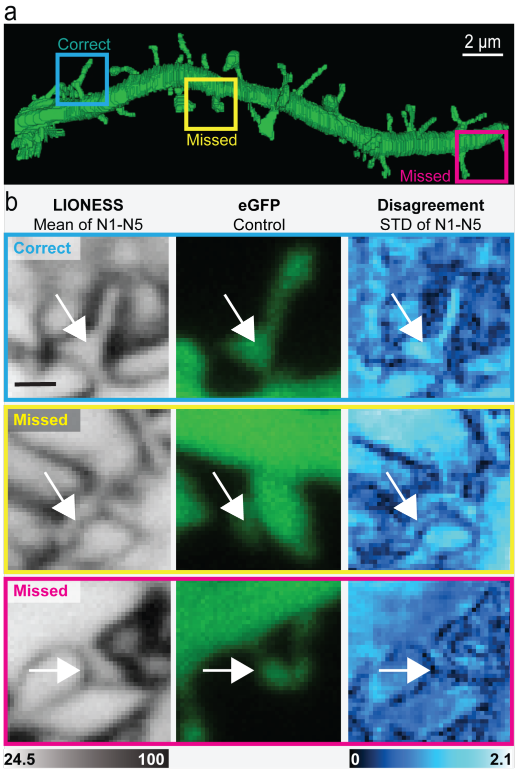

Supplementary Fig. 9| Spine detection errors in relation to ensemble disagreement in deep-learning based image restoration. a, Manual spine detection by segmenter blinded to intracellular eGFP signal (same data as in Fig. 2a). Coloured boxes indicate examples of correctly assigned and missed spines. **b,** Magnified views of boxed regions with 3D-super-resolved LIONESS data (*left*), intracellular eGFP signal recorded in confocal mode (*middle*), and ensemble disagreement (standard deviation, STD) at each voxel of 5 individual deep-learning image restoration networks N1-N5. Arrows indicate the location of spines taken as examples for the individual cases. LIONESS and prediction disagreement data are single planes whereas the eGFP signal is a maximum intensity projection across the whole image stack. Scale bar: 500 nm. Intensity values for LIONESS and disagreement are normalized to the maximum value in the LIONESS data. *Top*: Correctly detected spine despite comparatively high local ensemble disagreement (spine #35 in Fig. 2a). Note that the confocal eGFP signal is integrated over a larger axial range, thus showing also parts of the spine head that lie above or below the displayed LIONESS plane. *Middle* and *bottom*: Examples of spines that were overlooked by the segmenter despite lower values of ensemble disagreement at the respective locations (spines #25 and #5 in Fig. 2a). The images are representative of multiple occurrences of each case in the n=4 technical replicates displayed in Fig. 2a and Supplementary Fig. 8.

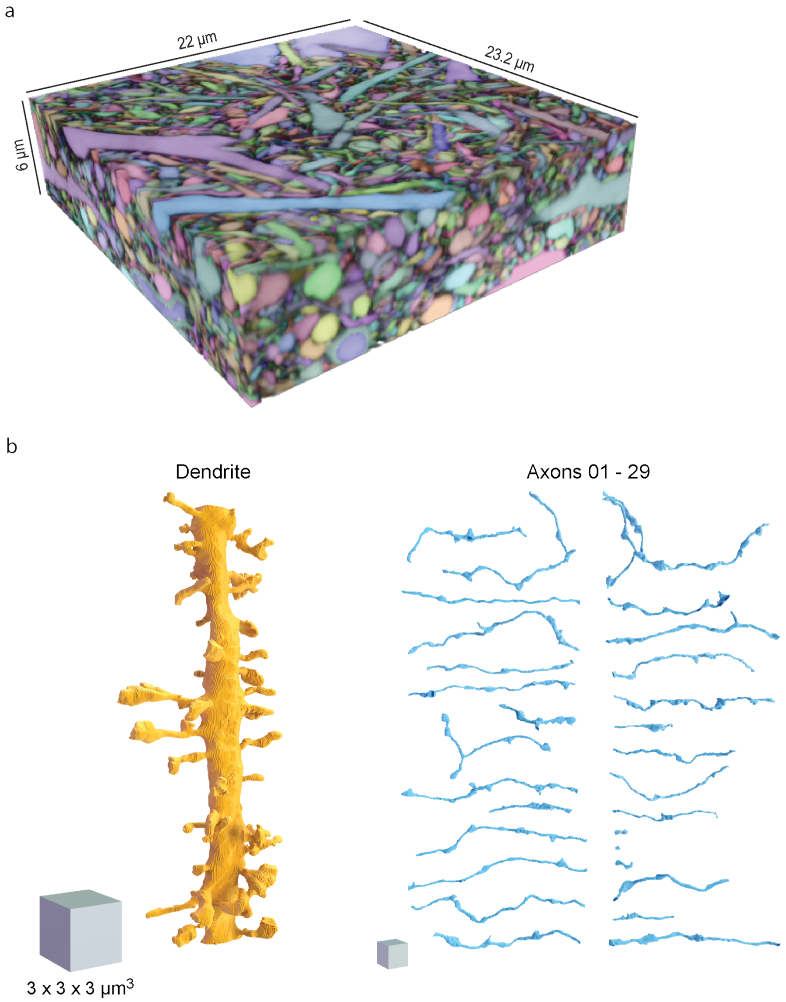

Supplementary Fig. 10| Reconstruction of spiny dendrite and putatively connected axons. a, The entire, automatically segmented dataset from Fig. 3, partially proofread. Volume dimensions: 23.2 x 22 x 6 μm^3. **b**, Spiny dendrite (gold) and the 29 individual putatively connected axons (blue) from Fig. 3. The dendrite is displayed without surface smoothing. Scale cubes refer to the center of the respective renderings and measure 3 x 3 x 3 μm^3. The two shortest axon segments correspond to one bouton at the edge of the imaging volume and one bouton which could not be unambiguously assigned to an axon.

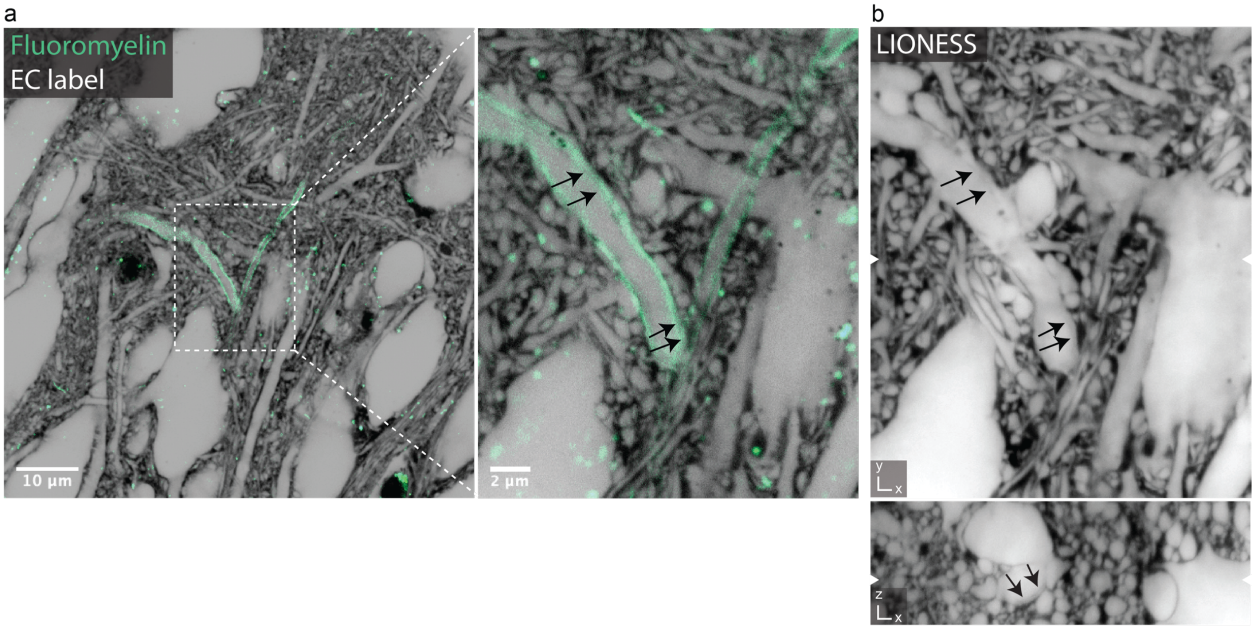

Supplementary Fig. 11| Identification of myelinated axons. a, *Left*: Confocal overview image in organotypic hippocampal slice culture with extracellular (grey, inverted intensity lookup table) and additional myelin labelling (Fluoromyelin, green). *Right*: Magnified view highlighting a myelinated axon. Single plane in isotropically resolving STED mode for extracellular label and confocal mode for the myelin stain. Scale bars: 10 µm (left), 2 µm (right). **b**, Volumetric LIONESS acquisition of the same region. Black arrows indicate the border between axon and myelin sheath visible in the LIONESS data. White arrowheads at image edges indicate the corresponding position of *xy*- and *xz*-views. The images are representative for n=3 technical replicates recorded across 2 different biological specimens. LIONESS images are maximum intensity projections spanning 150 nm.

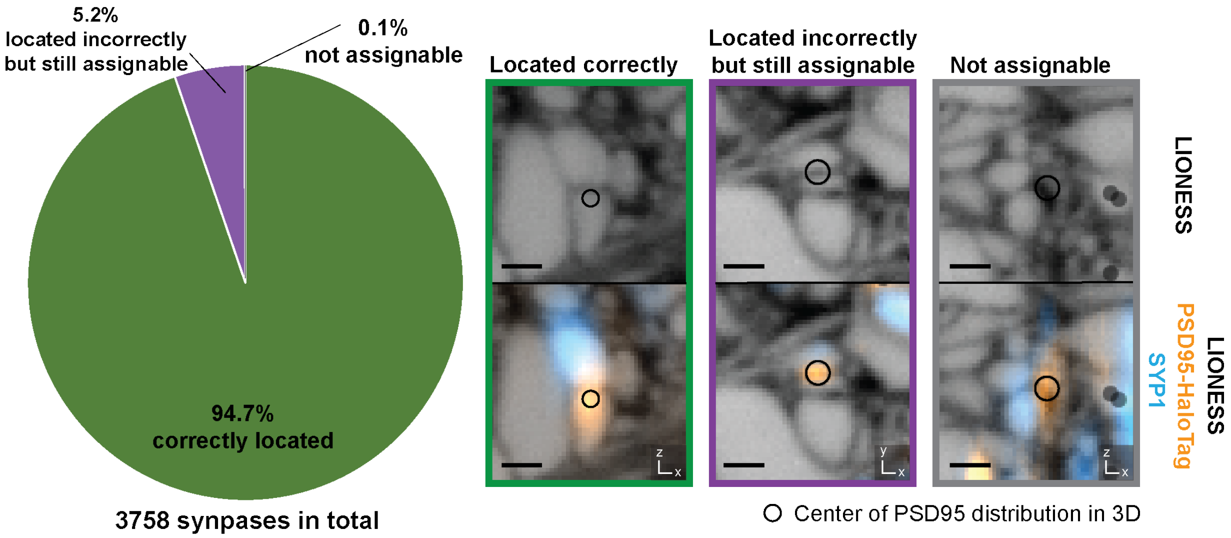

Supplementary Fig. 12| Assignment of molecular information to synaptic structures. Localization of confocal PSD95 signal with respect to 3D super-resolved LIONESS data. *Left*: Quantification of PSD95 assignment to synaptic structures for 3758 synapses recorded across n=3 different organotypic hippocampal slices generated from *PSD95-HaloTag* transgenic mice. Data acquisition was identical to Fig. 4. *Right*: Example images with corresponding color coding. *Top*: Single planes of LIONESS data. Open circles indicate the center of the 3D distribution of the PSD95 signal as determined by the Laplacian of Gaussian (LoG) detector of the FIJI TrackMate plugin. Closed circles indicate positions of further PSD95 signals in different imaging planes. *Bottom*: Overlay of LIONESS data with PSD95 and SYP1 confocal signals. Scale bars: 500 nm. Molecular signals were classified as "correctly located" (green frame) if the center of the PSD95 signal (black circle) was located within the postsynaptic structure in 3D. The postsynaptic structure was defined by morphology and, where available, additionally taking the presynaptic synaptic vesicle staining (SYP1, confocal) into account. PSD95 signals that were not located inside a postsynaptic structure but still assignable using morphology and additional SYP1 labeling were classified as "located incorrectly, still assignable" (purple frame). A few signals were not assignable to any structure due to ambiguities in the LIONESS data (gray frame). The images are representative of n=3 measurements recorded across 2 biological specimens.

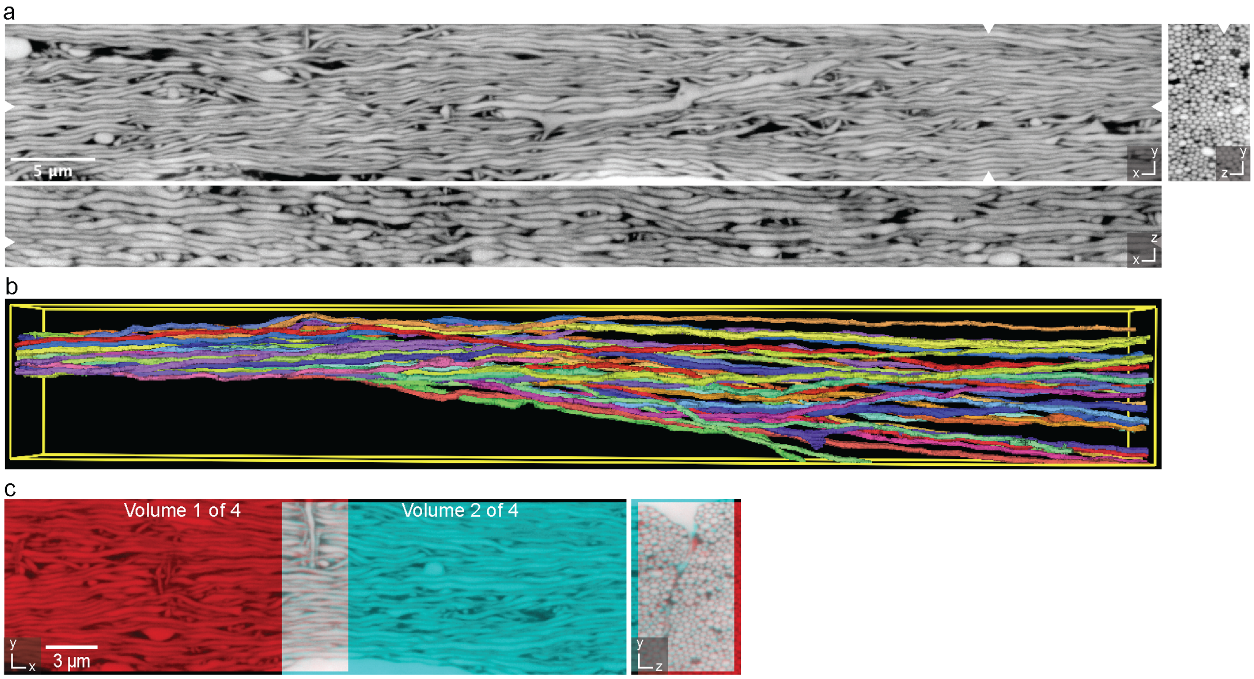

Supplementary Fig. 13| Extending LIONESS tissue volumes. a, Orthogonal planes in *xy*-, *xz*-, and *yz*-directions from a LIONESS volume in the alveus region of an acutely prepared mouse hippocampus. Data were registered from 4 consecutive, partially overlapping super-resolved acquisitions. White arrowheads at image edges indicate position of corresponding orthogonal planes. Maximum intensity projections spanning 150 nm. LIONESS imaging in the alveus region of acutely prepared hippocampus is representative of imaging in n=4 biological specimens. Scale bar: 5 µm. **b**, 3D-rendering of selected axons from panel a, forming a tight bundle in the left and progressively fanning out. **c**, Example of alignment between two of the partially overlapping subvolumes in *xy*- and *yz*-views. Individual subvolumes are shown in red and cyan, such that overlapping regions add up to white color, indicating the degree of overlap.

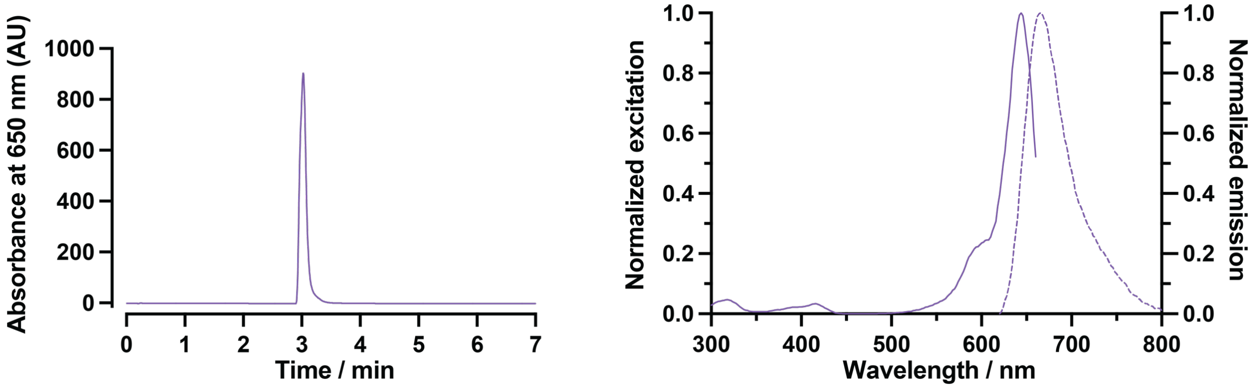

Supplementary Fig. 14| Characterization of SulfoAtto 643. *Left*: Absorbance as a function of elution time acquired by liquid chromatography low resolution mass spectrometry. *Right*: Normalized excitation and emission spectra of SulfoAtto 643.

Supplementary References

1. Feng, G. P. *et al.* Imaging neuronal subsets in transgenic mice expressing multiple spectral variants of GFP. *Neuron* **28**, 41–51 (2000).
