# Supplementary material for: Dense 4D nanoscale reconstruction of living brain tissue
Source: Nat Methods. 2023 Jul 10;20(8):1256–65. doi: 10.1038/s41592-023-01936-6 (PMC10406607; doi:10.1038/s41592-023-01936-6)
Supplement: Supplementary file 2 — Reporting Summary [file 41592_2023_1936_MOESM2_ESM.pdf]

nature portfolio

Corresponding author(s): Johann G. Danzl

Last updated by author(s): 2023/06/28

Reporting Summary

Nature Portfolio wishes to improve the reproducibility of the work that we publish. This form provides structure for consistency and transparency in reporting. For further information on Nature Portfolio policies, see our Editorial Policies and the Editorial Policy Checklist.

Statistics

For all statistical analyses, confirm that the following items are present in the figure legend, table legend, main text, or Methods section.

n/a	Confirmed	
☐	☒	The exact sample size (*n*) for each experimental group/condition, given as a discrete number and unit of measurement
☐	☒	A statement on whether measurements were taken from distinct samples or whether the same sample was measured repeatedly
☒	☐	The statistical test(s) used AND whether they are one- or two-sided<br>*Only common tests should be described solely by name; describe more complex techniques in the Methods section.*
☒	☐	A description of all covariates tested
☒	☐	A description of any assumptions or corrections, such as tests of normality and adjustment for multiple comparisons
☐	☒	A full description of the statistical parameters including central tendency (e.g. means) or other basic estimates (e.g. regression coefficient) AND variation (e.g. standard deviation) or associated estimates of uncertainty (e.g. confidence intervals)
☒	☐	For null hypothesis testing, the test statistic (e.g. *F*, *t*, *r*) with confidence intervals, effect sizes, degrees of freedom and *P* value noted<br>*Give P values as exact values whenever suitable.*
☒	☐	For Bayesian analysis, information on the choice of priors and Markov chain Monte Carlo settings
☒	☐	For hierarchical and complex designs, identification of the appropriate level for tests and full reporting of outcomes
☒	☐	Estimates of effect sizes (e.g. Cohen's *d*, Pearson's *r*), indicating how they were calculated

Our web collection on statistics for biologists contains articles on many of the points above.

Software and code

Policy information about availability of computer code

Data collection	Data were collected using an Abberior Instruments Expert Line STED microscope using Imspector software (version 14.0.3052 and 16.3.13031).
Data analysis	Image analysis was done in Fiji (Version 2.3.0/1.53f) including cell counter, calculator plus, 3D stitching, and TrackMate v7.6.0 plugins. Deep learning-based image restoration and image de-noising were done using CSBDeep 0.6.1 (https://github.com/CSBDeep/CSBDeep) and noise2void (Version 0.2.1, https://github.com/juglab/n2v), respectively. Manual segmentation and proofreading were done with VAST v1.3.0, v1.4.0 and v1.4.1. Automatic segmentation was based on the pytorch_connectomics framework (arXiv preprint arXiv:2112.05754) followed by application of two watershed algorithms (https://github.com/zudi-lin/zwatershed and https://github.com/zudi-lin/waterz). Custom code is provided as Supplementary Software zip file and is available via https://github.com/danzllab/LIONESS. Manual tracing was done with WebKnossos v22.05.1. For statistical analysis and plotting Microsoft Excel for Mac (Version 16.59) and GraphPad Prism (Version 9.0.2) were used. Blender v2.93.4, VAST v1.4.0, Neuroglancer and Neuromorph 2.8 was used for visualization. 3D meshes for visualization were generated from segmentations using Scikit-Image, Python v2.7 or v3.7.12. Electrophysiological recordings were analysed with Stimfit (Front Neuroinform 8, 16 (2014)) and MATLAB-based scripts. Supplementary movies were generated with iMovie. The schematics in the upper row of Fig. 1a were generated with Biorender.com. Denderite abstraction in Fig. 3e was based on Barrio (Computer Graphics Forum 41, 183–194 (2022)).

For manuscripts utilizing custom algorithms or software that are central to the research but not yet described in published literature, software must be made available to editors and reviewers. We strongly encourage code deposition in a community repository (e.g. GitHub). See the Nature Portfolio guidelines for submitting code & software for further information.

Data

Policy information about availability of data

All manuscripts must include a data availability statement. This statement should provide the following information, where applicable:

- Accession codes, unique identifiers, or web links for publicly available datasets
- A description of any restrictions on data availability
- For clinical datasets or third party data, please ensure that the statement adheres to our policy

Imaging data and models are available at the Institute of Science and Technology Austria's data repository with DOI: 10.15479/AT:ISTA:12817 (https://research-explorer.ista.ac.at/record/12817).

Field-specific reporting

Please select the one below that is the best fit for your research. If you are not sure, read the appropriate sections before making your selection.

☒ Life sciences ☐ Behavioural & social sciences ☐ Ecological, evolutionary & environmental sciences

For a reference copy of the document with all sections, see nature.com/documents/nr-reporting-summary-flat.pdf

Life sciences study design

All studies must disclose on these points even when the disclosure is negative.

Sample size

The manuscript reports on a technological development. No biological conclusions are drawn in the paper. Experimental replicates were performed to demonstrate technical reproducibility and not to describe any biological variability. Accordingly, no statistical methods to predetermine sample size for biological specimens were applied. Technical replicates were either carried out in the same biological specimen or across multiple specimens, as indicated under "Replication".

Data exclusions

Some images or image volumes displayed are cropped from larger raw data, focusing on the region of best optical quality in the centre of the objective's field of view and optionally specific regions of interest. Smaller subvolumes also facilitated data handling. For LIONESS imaging and in silico reconstruction, optimum sample preparation, sample mounting, and microscope alignment conditions as well as imaging parameters were required. Datasets of lower quality were not further analysed.

Replication

LIONESS imaging of cerebral organoids as depicted in Fig. 1a and Supplementary Fig. 6,7 was additionally repeated on similar specimens twice (n=3) and the LIONESS volume displayed in the figures was selected for reconstruction. The direct juxtaposition of STED and LIONESS for the same STED light parameters in Fig. 1b,c was performed in n=3 technical replicates from 2 samples. The images in Fig. 2a stem from one dataset and the data in Fig. 2b correspond to n=4 technical replicates (i.e. different LIONESS imaging volumes containing a positively labelled dendrite stretch). These were recorded from a total of 3 different biological specimens (i.e. 3 different organotypic brain slices), with the additional datasets displayed in Supplementary Fig. 8. The images in Fig. 2c-e are representative of tracing 9 axons in a total of n=3 biological replicates. LIONESS imaging in neuropil of organotypic hippocampal slice cultures as in Fig. 3, Extended Data Fig. 1 and 10 and Supplementary Fig. 1, 4, and 10 was repeated over 20 times. Proofreading of automated segmentation and 3D-visualization and analysis in Fig. 3b-e and Supplementary Fig. 10 was applied to one dataset. LIONESS imaging paired with PSD95-HaloTag/SYP1-EGFP live labelling as in Fig. 4a,b and Extended Data Fig. 5 is representative of experiments in n=4 different biological samples. Proofreading of automated reconstruction and 3D-visualization in Fig. 4b was performed for one specimen. LIONESS imaging in DREADD expressing samples in Fig. 5 was done in two biological replicates and proofreading of the automated segmentation and 3D visualization were performed in one of these.
Measurements of point spread functions on gold beads (Extended Data Fig. 1a,b) were performed for routine microscope alignment (n>20) and the measurements of effective point spread function on fluorescent beads in Extended Data Fig. 1c are representative of n=2 repetitions. The direct comparison of performance in confocal and STED imaging with the indicated phase modulation patterns in Extended Data Fig. 1d is representative of n=3 technical replicates recorded in two biological samples. Direct comparison of conventional STED light exposure and LIONESS in Extended Data Fig. 2 is representative of n=4 experiments from 2 biological samples. Here, repeated exposure of the same region with conventional, high photon load STED (Extended Data Fig. 2a) was reproduced with performing xy-scanning only, showing the same negative effect. In Extended Data Fig. 3a, we excluded one dataset (n=1) from the image restoration training for testing whereas 75 volumes were included in the training. Images in Extended Data Fig. 3b are taken from n=5 technical replicates recorded across 4 biological specimens. Acute preparation of hippocampus and LIONESS imaging of the alveus region as shown in Extended Data Fig. 4 and Supplementary Fig. 13 was repeated in n=4 samples and the respective segmentations and analyses were performed on the two examples selected for display. Repeated LIONESS imaging of the same sample volume at various timings was performed in more than 4 samples. Of these, datasets in Fig. 5, Extended Data Fig. 2b, 6, 8 were selected for the respective figures to demonstrate specific timing aspects. Imaging over 3 days in Extended Data Fig. 6 was done in one sample. The 5 spines segmented at 2 time points in Extended Data Fig. 7 were from n=2 independent samples. The data on LIONESS combined with Ca2+ imaging in Extended Data Fig. 8 are representative of n=4 technical replicates recorded across 3 different biological samples. The data on combining LIONESS with patch clamp recordings in Extended Data Fig. 9 are representative of n=3 biological replicates.
The images in Supplementary Fig. 1b are representative for a large number of measurements (n>>20), as we performed all our LIONESS imaging with these fluorophores. In contrast, we discarded fluorophores that either exhibited poor STED performance or entered cells after n=2 experiments (Supplementary Fig. 1a). Comparison of single versus split detection (Supplementary Fig. 2) is representative for n=3 technical replicates in the same specimen. We performed all experiments within the region of optimum imaging performance (~25x25 µm2 laterally and ~8-10 µm axially, up to a depth of ~50 µm). The visualization of performance outside this region in Supplementary Fig. 3a,b is representative of n=2 technical replicates in the same sample. For testing voxel based uncertainty measures in Supplementary Fig. 4b, we excluded one dataset (n=1) from the image restoration training for testing, whereas 75 volumes were included in the training. Manual

segmentation in neuropil of an organotypic hippocampal slice culture (Supplementary Fig. 5) was done in n=1 dataset. The data in Supplementary Fig. 9 represents the same dataset as Fig. 2a. Displayed examples for correctly identified and missed spines are representative of multiple occurrences of these cases in the n=4 technical replicates of this measurement (see Fig. 2a and Supplementary Fig. 8). FluoroMyelin imaging as shown in Supplementary Fig. 11 was performed in n=3 brain slices from two mice and the assignment of synaptic proteins in Supplementary Fig. 12 contained 3758 synapses recorded in n=3 measurements from two specimens.

Randomization

We do not compare samples between experimental groups. Accordingly, no randomization was performed.

Blinding

The neuroscientist who performed manual segmentation of dendrites and the tracing of axons in Fig. 2 was blinded to the positively labelled (GFP) ground truth images.
In Fig. 1,3,4,5, we characterize the technology based on imaging data and visualizations. In the Extended Data Figures and Supplementary Figures, we give more technical details and imaging data. Blinding would not be useful or possible for such experiments.

Reporting for specific materials, systems and methods

We require information from authors about some types of materials, experimental systems and methods used in many studies. Here, indicate whether each material, system or method listed is relevant to your study. If you are not sure if a list item applies to your research, read the appropriate section before selecting a response.

Materials & experimental systems

n/a	Involved in the study
☒	☐ Antibodies
☐	☒ Eukaryotic cell lines
☒	☐ Palaeontology and archaeology
☐	☒ Animals and other organisms
☒	☐ Human research participants
☒	☐ Clinical data
☒	☐ Dual use research of concern

Methods

n/a	Involved in the study
☒	☐ ChIP-seq
☒	☐ Flow cytometry
☒	☐ MRI-based neuroimaging

Eukaryotic cell lines

Policy information about cell lines

Cell line source(s)

For generation of human cerebral organoids, human H9 ES cells (WA09, https://hpscreg.eu/cell-line/WAe009-A) were obtained from a commercial source (WiCell). Research involving generation of cerebral organoids from these cells was approved by IST Austria's institutional ethics board (ISTA Ethics Committee, approval date June 09, 2020). HEK293T were purchased from ATCC (CRL-3216). HEK-GT and BHK-eT were previously generated at ISTA and described in Sumser et al., https://doi.org/10.7554/eLife.79848.

Authentication

Commercial H9 ES and HEK293T cell lines were authenticated by the provider. No further authentication was performed.

Mycoplasma contamination

Cells were routinely tested for Mycoplasma contamination and were tested negative.

Commonly misidentified lines
(See ICLAC register)

The study did not involve commonly misidentified cell lines.

Animals and other organisms

Policy information about studies involving animals; ARRIVE guidelines recommended for reporting animal research

Laboratory animals

Animals were housed in groups of 3-4 animals under controlled laboratory conditions (12:12 h light/dark cycle with lights on at 07:00 hours; 21 ± 1 °C; 55 ± 10 % humidity) with food (pellets, 10 mm) and autoclaved water ad libitum. The animals were housed in commercially available individually ventilated cages (IVCs) made from Polysulfon with a solid cage floor, dust free bedding (woodchips) and nesting material.
Organotypic slice cultures and acutely prepared hippocampus were generated from 5-7 day old mice, including wild-type C57BL/6J animals and the following transgenic strains: B6;129S-Gt(ROSA)26Sortm95.1(CAG-GCaMP6f)Hze/J (Jackson #024105), Tg(Prox1-cre)SJ32Gsat/Mmucd (MMRRC #036644-UCD, PSD95-HaloTag (Seth G.N. Grant, Edinburgh University) and STOCK Tg(Thy1-EGFP)MJrs/J (Jackson #007788). Mice of either sex were used interchangeably for tissue culture and acute tissue preparation.

Wild animals

The study did not involve wild animals.

Field-collected samples

The study did not involve samples collected from the field.

Ethics oversight

Procedures were performed in accordance with national law (BGBLA 114 and Directive 522), European Directive 2010/63/EU and institutional guidelines for animal experimentation.
Experiments were performed on organotypic hippocampal slice cultures and acutely prepared hippocampus. This involves organ extraction after euthanizing the animal, which does not require ethics approval.

Note that full information on the approval of the study protocol must also be provided in the manuscript.
