# Supplementary figures and images for: Dense 4D nanoscale reconstruction of living brain tissue
Source: Nat Methods. 2023 Jul 10;20(8):1256–65. doi: 10.1038/s41592-023-01936-6 (PMC10406607; doi:10.1038/s41592-023-01936-6)

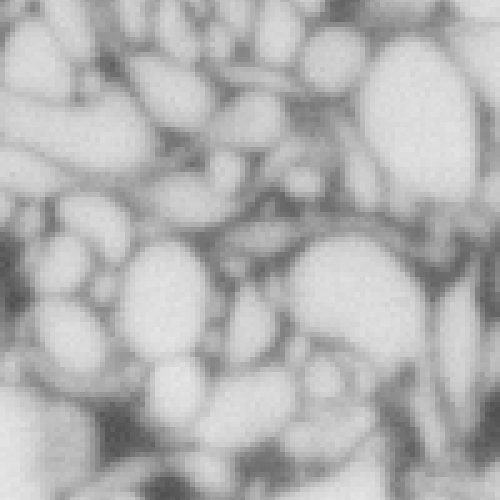

Supplement: Supplementary file 13 — Zip file containing custom software/code. [file 41592_2023_1936_MOESM13_ESM.zip › LIONESS/demo_data/lioness_data.tif]

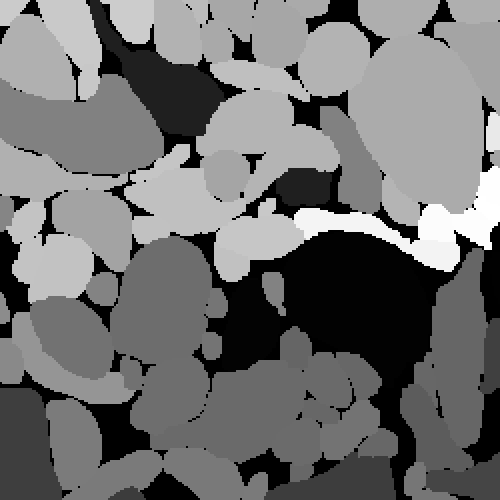

Supplement: Supplementary file 13 — Zip file containing custom software/code. [file 41592_2023_1936_MOESM13_ESM.zip › LIONESS/demo_data/segmentation.tif]
